# A flexible symbolic regression method for constructing interpretable clinical prediction models

**DOI:** 10.1038/s41746-023-00833-8

**Published:** 2023-06-05

**Authors:** William G. La Cava, Paul C. Lee, Imran Ajmal, Xiruo Ding, Priyanka Solanki, Jordana B. Cohen, Jason H. Moore, Daniel S. Herman

**Affiliations:** 1 Computational Health Informatics Program, Boston Children’s Hospital, Harvard Medical School, Boston, MA USA; 2 Department of Pathology and Laboratory Medicine, University of Pennsylvania, Philadelphia, PA USA; 3 Division of Renal-Electrolyte and Hypertension, Department of Medicine, University of Pennsylvania, Philadelphia, PA USA; 4 Department of Biostatistics, Epidemiology, and Informatics, University of Pennsylvania, Philadelphia, PA USA

**Keywords:** Diagnosis, Computational science, Hypertension, Statistics

## Abstract

Machine learning (ML) models trained for triggering clinical decision support (CDS) are typically either accurate or interpretable but not both. Scaling CDS to the panoply of clinical use cases while mitigating risks to patients will require many ML models be intuitively interpretable for clinicians. To this end, we adapted a symbolic regression method, coined the feature engineering automation tool (FEAT), to train concise and accurate models from high-dimensional electronic health record (EHR) data. We first present an in-depth application of FEAT to classify hypertension, hypertension with unexplained hypokalemia, and apparent treatment-resistant hypertension (aTRH) using EHR data for 1200 subjects receiving longitudinal care in a large healthcare system. FEAT models trained to predict phenotypes adjudicated by chart review had equivalent or higher discriminative performance (*p* < 0.001) and were at least three times smaller (*p* < 1 × 10^−6^) than other potentially interpretable models. For aTRH, FEAT generated a six-feature, highly discriminative (positive predictive value = 0.70, sensitivity = 0.62), and clinically intuitive model. To assess the generalizability of the approach, we tested FEAT on 25 benchmark clinical phenotyping tasks using the MIMIC-III critical care database. Under comparable dimensionality constraints, FEAT’s models exhibited higher area under the receiver-operating curve scores than penalized linear models across tasks (*p* < 6 × 10^−6^). In summary, FEAT can train EHR prediction models that are both intuitively interpretable and accurate, which should facilitate safe and effective scaling of ML-triggered CDS to the panoply of potential clinical use cases and healthcare practices.

## Introduction

Interpretable machine learning (ML) models are essential to realizing the potential for systematic, targeted clinical decision support (CDS) to radically transform the practice of medicine. Most CDS is currently triggered based on manually curated rules or heuristics that identify patient cohorts with certain characteristics of interest^1–3^. Such approaches can achieve high accuracy, but in the setting of complex, imprecise, and heterogeneous clinical phenotypes and high-dimensional electronic health record (EHR) data, curating such rules requires considerable time and expertise^4–9^. The effort needed for such approaches impedes their scalability to widespread, effective CDS.

Recent advances in ML and the ever-improving availability of EHR data herald the use of ML for systematic, targeted CDS^4,5^. While the potential gains are massive, the challenges are also considerable. Realizing systematic CDS requires learning and deploying highly accurate clinical prediction models for a myriad of clinical phenotypes in the setting of considerable variability in clinical practice and documentation across providers and health systems. Because of these challenges, and particularly because of the novelty of the tools and approaches, it is essential to comprehensively assess and balance the potential benefits and risks of each model-triggered CDS intervention.

One major determinant of the risk of a model-triggered intervention is the model’s *interpretability*^10–13^. Interpretability, which is related to explainability, is a somewhat subjective concept that we will use to describe whether the user, here a clinical practitioner, can understand how and *why* the model is calling an individual patient positive or negative^13,14^. Interpretable models are more naturally incorporated within existing decision-making frameworks whereby clinicians can corroborate or second-guess predictions, ultimately leading to trust and yielding overall higher quality decisions. For these reasons, the FDA’s proposed regulatory framework for the evaluation of automated clinical decision support systems incorporates interpretability as part of its risk stratification, described as whether clinicians can “independently review the basis for [a model’s] recommendations”^15^. There is much debate about the importance of model interpretability for deploying safe and effective ML-based CDS^16–18^. While all models need not be interpretable^19^, interpretable models are preferable if they have comparable performance or if a modest cost to performance is outweighed by lower risk or better incorporation into clinical practice that ultimately yields greater overall clinical utility.

Expert-curated heuristics are inherently interpretable, but the majority of ML models learned for CDS have limited interpretability^20–27^. Post-hoc approaches typically estimate the impact of individual features for particular samples or sets of samples, but these approximations do not yield complete interpretability or accuracy, particularly when a model’s components are intricate or high-dimensional^16,28–31^. We believe this lack of interpretability significantly delays the deployment of ML-based CDS. To achieve trustworthy explanations, researchers may turn to well-understood and transparent ML approaches such as (penalized) linear models, but often do so at the expense of discriminative performance.

**Fig. 1:**
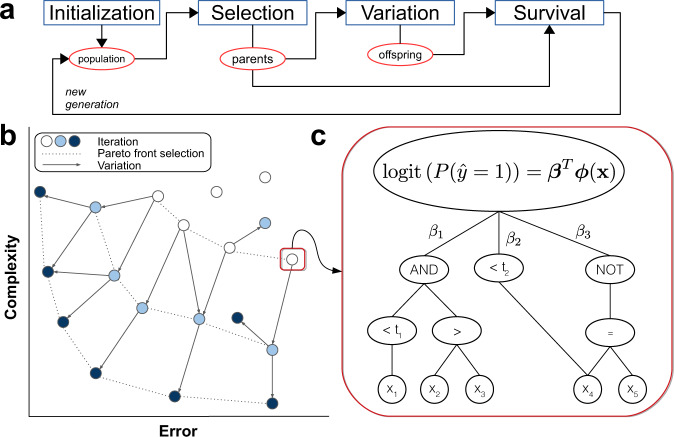
How FEAT works. **a** Steps in the genetic programming process. Candidate models are initialized in a population; the best models (parents) are selected via epsilon-lexicase selection; offspring are created by applying variation operations to the parents; and then parents and offspring compete in a survival step using NSGA-II [22]. The process then repeats. **b** The evaluation of a candidate models’ complexity and performance in Pareto Optimization framework in the Survival step. **c** Example model in which input features are transformed by logical functions with or without threshold operators.

In an effort to train EHR prediction models that are both accurate and interpretable, we adapted and further developed the feature engineering automation tool (FEAT)^32–34^. FEAT is a symbolic regression method^35^ that searches for simple, interpretable feature representations in tandem with fitting a classification model (Fig. 1). The representations are trained using a population-based Pareto optimization algorithm that jointly optimizes model discrimination and complexity^36,37^. To our knowledge, this is the first work to explore the application of symbolic regression with Pareto optimization to EHR prediction modeling.

We applied FEAT to EHR data targeting screening for primary aldosteronism (PA), the most frequent cause of secondary hypertension^38^. PA appears to affect up to ~1% of US adults and it is highly treatable; unfortunately, it is under-screened for and under-diagnosed^39–44^. Multiple specialty society guidelines recommend PA screening for patients with hypertension with unexplained hypokalemia (HTN-hk) or apparent treatment-resistant hypertension (aTRH) as PA is thought to be responsible for these phenotypes in up to 20% of affected patients^39–41,45^. We have applied FEAT to learn EHR prediction models for identifying patients who should be screened for PA. For this clinical goal, interpretable models are highly advantageous because these clinical phenotypes are complex and there is considerable variability in diagnosis and management across practitioners. Using an interpretable prediction, clinicians should be able to evaluate the models’ results and then make their own model prediction-informed decision. We expect that identifying patients that meet PA screening guidelines in such a manner will help providers improve their management of these patients, leading to better blood pressure control and ultimately decreased cardiovascular morbidity and mortality.

We evaluated FEAT’s ability to generate interpretable and accurate models in comparison to other potentially “white-box” or “glass-box” approaches, including penalized logistic regression, as well as in comparison to commonly used “black-box” methods random forests and neural networks. FEAT-trained models were first compared for three increasingly complex phenotypes that are components of PA screening criteria, adjudicated by chart review. To explore the generalizability of the method, we then applied FEAT to a panel of 25 open, previously studied clinical prediction tasks in MIMIC-III^46^. We found that FEAT was able to generate considerably simpler models than traditional “white-box” approaches and achieved equivalent or better accuracy. FEAT’s models were also much smaller than black-box approaches (random forest and long-short-term memory networks (LSTMs)), which demonstrated only slightly higher discrimination for several but not all of the phenotypes. These results demonstrate the promise of symbolic regression for generating clinical prediction models that are both accurate and interpretable.

## Results

### Development of a symbolic regression method FEAT for constructing accurate and interpretable EHR prediction models

To train EHR prediction models whose outputs are intuitively interpretable by clinicians, we adapted and further developed the ML tool FEAT (Fig. 1) to better implement Boolean logic, added procedures to encourage model parsimony, and developed approaches for improving training robustness. We evaluated the modifications to FEAT (listed in Supplementary Table 1) on 20 benchmark classification problems from the Penn Machine Learning Benchmark (PMLB)^47^ that were similar in shape to our PA screening EHR data (Supplementary Table 2). Unless otherwise noted, the statistical tests reported below are based on Wilcoxon rank-sum tests.

We found that restricting operators and simplifying models decreased the size of resulting models by more than two-fold (*p* = 7.2 × 10^−9^) without substantially impairing classification performance (Supplementary Fig. 1). We considered restricting FEAT to produce models with only a single derived feature (i.e. Feat_1dim). While this constraint further decreased median model size by 71% (*p* = 1.4 × 10^−18^), these models demonstrated markedly lower AUPRC (*p* = 1.5 × 10^−4^). Therefore, in this work we used the version of FEAT that included feature representation simplification components and a restricted operator set but allowed multiple derived features (i.e., FEAT_boolean_simplify).

### Automated learning of EHR prediction models

We next applied our optimized FEAT method to a training dataset of 899 subjects to learn to replicate heuristics that were expert-curated to identify patients with hypertension, HTN-hk, and aTRH. We compared the constructed models to those trained by other ML methods that have the potential to build interpretable models: LASSO-penalized logistic regression (LR L1), ridge-penalized logistic regression (LR L2), decision trees (DT), and Gaussian Naïve Bayes (GNB). Across all three heuristics, FEAT models achieved higher area under the precision-recall curve (AUPRC) (range: +0.01 to +0.52; *p* < 0.001) than all other models and were smaller than all other models (range: 3-fold to 40-fold; *p* < 1 × 10^−6^) except DT models (Supplementary Figs. 2 & 3, Supplementary Table 3).

**Fig. 2:**
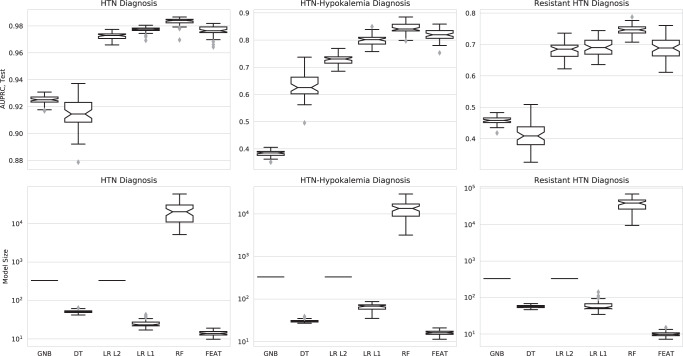
Estimating model discrimination and size by cross-validation. (Top row) AUPRC scores for phenotyping models trained in 5-fold cross-validation over 50 iterations, each averaged across testing folds. (Bottom row) Sizes of the trained models. Each subplot represents a different training outcome, as determined by chart review. Box centerline: median; box limits: quartiles; whiskers: 1.5x the interquartile range; diamonds: outliers.

**Fig. 3:**
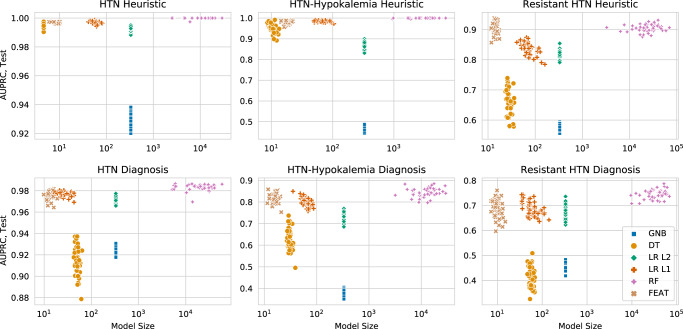
The tradeoff between model discrimination and complexity. Each point shows the cross-validation testing AUPRC (y-axis) and size (x-axis) for models trained in 50 repeat trials for each method. Each subplot represents a different expert-curated heuristic (top row) or chart review phenotype (bottom row). The ideal model is discriminative and simple, meaning it is near the top left corner. DT models occupy this zone for modeling simple heuristics, whereas FEAT models tend to be simple and accurate across all experiments.

**Fig. 4:**
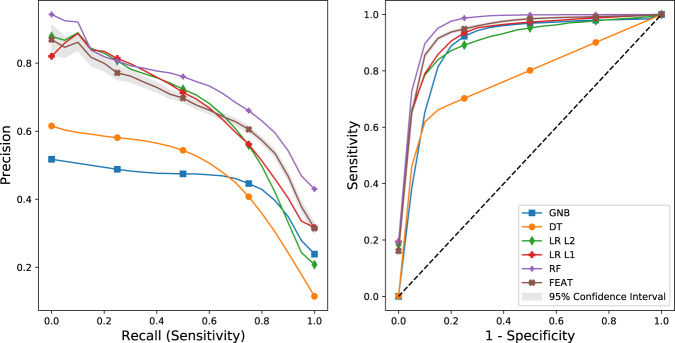
Model precision-recall and receiver-operating curves. Precision-recall curves (left) and receiver-operating curves (right) for phenotyping models trained to predict chart review classifications for aTRH. Values shown are means of test performance in 5-fold cross-validation repeated 50 times.

Next, we compared these methods’ abilities to train models to predict more complicated realizations of these phenotypes as assessed by chart review, which were present in 423 (47%), 93 (10%), and 103 (11%) subjects, respectively. Across all phenotypes, FEAT models achieved higher AUPRC scores than GNB (range: +0.05 to +0.44; *p* < 0.001), DT (range: +0.07 to +0.28; *p* < 0.001), and LR L2 (range: +0.00 to +0.09; *p* < 0.001) models and similar AUPRC to LR L1 (range: 0.00 to +0.02) models (Fig. 2; Supplementary Fig. 3 and Table 4). At the same time, FEAT models were considerably smaller (range: 1.7-fold to 34-fold; *p* < 1e–6) than *all* other models including DT models. We next explored the trade-off between model performance and complexity for heuristic and chart-review trained models (Fig. 3). The FEAT models clustered near the high-AUPRC, low-complexity region (top left) of this tradeoff space, indicating that relative to the other methods they consistently achieved an efficient trade-off between these two performance objectives. Focusing on the most complex phenotype, aTRH by chart review, FEAT models achieved a median AUPRC of 0.69 (interquartile range [IQR]: 0.05) with a median size of 9.8 (IQR: 1.8). These models showed reasonable discrimination across a broad range of potential decision thresholds, as depicted by AUPRC and area under the receiver-operating curve (AUROC) in Fig. 4.

We next compared FEAT models to random forests (RF) models, a representative black-box method. As expected, FEAT models were orders of magnitude smaller than the RF-trained models (Figs. 2 & 3, Supplementary Tables 3 & 4). FEAT models showed similar AUPRC for all heuristics as well as for hypertension and HTN-hk by chart review and a slightly lower AUPRC (0.69 ± 0.05 versus 0.75 ± 0.02) for aTRH by chart review.

### Final model training and evaluation

Next, we applied the methods refined by cross-validation to train models on the entire training set and assessed their performance on a held-out test set of 300 subjects, including 185 (61%), 79 (26%), and 73 (24%) subjects positive for each chart-review phenotype. Model performance and size (Table 1) were largely consistent with cross-validation estimates, although most appeared to have slightly better AUPRC likely due in part to the greater enrichment for heuristic-ascertained cases in the testing cohort. For chart-review hypertension, HTN-hk, and aTRH, the FEAT models demonstrated AUPRC scores of 0.99 (95% CI: ±0.01), 0.96 ( ± 0.04), and 0.78 ( ± 0.08), and AUROC scores of 0.99 ( ± 0.01), 0.98 ( ± 0.02), and 0.93 ( ± 0.03), respectively. Across phenotypes, the FEAT models were considerably smaller than those produced by the other potentially interpretable modeling methods and their AUPRC and AUROC scores were comparable to that of the most discriminative model (Table 1).

**Table 1:** Final model discrimination in test set and size by chart-review phenotype.

Phenotype	Method	Test AUPRC	Test AUROC	Model Size
HTN	GNB	0.97 (0.94–0.98)	0.96 (0.94–0.98)	331
	DT	0.97 (0.95–0.99)	**0.97 (0.95–0.99)**	43
	LR L1	**1.0 (0.99–1.0)**	**0.99 (0.99–1.0)**	32
	LR L2	**0.99 (0.99–1.0)**	**0.98 (0.97–0.99)**	331
	RF	**1.0 (0.99–1.0)**	**0.99 (0.99–1.0)**	67,276
	FEAT	**0.99 (0.98–1.0)**	**0.99 (0.98–1.0)**	**18**
HTN-Hypokalemia	GNB	0.61 (0.52–0.69)	0.81 (0.76–0.86)	331
	DT	0.75 (0.67–0.82)	0.86 (0.81–0.9)	33
	LR L1	**0.95 (0.92–0.97)**	**0.98 (0.96–0.99)**	29
	LR L2	**0.92 (0.88–0.95)**	**0.96 (0.93–0.98)**	331
	RF	**0.96 (0.93–0.99)**	**0.99 (0.98–0.99)**	16,256
	FEAT	**0.96 (0.93–0.98)**	**0.98 (0.96–0.99)**	**8**
Resistant HTN	GNB	0.57 (0.49–0.66)	0.86 (0.82–0.89)	331
	DT	0.24 (0.19–0.28)	0.47 (0.41–0.52)	67
	LR L1	0.74 (0.66–0.82)	0.87 (0.83–0.91)	130
	LR L2	**0.78 (0.69–0.86)**	0.91 (0.87–0.94)	331
	RF	**0.90 (0.84–0.94)**	**0.96 (0.95–0.98)**	119,760
	FEAT	**0.78 (0.69–0.85)**	**0.93 (0.91–0.95)**	**11**

Bootstrapped 95% confidence intervals (CIs) shown in parenthesis. Bold indicates best models and those with an overlapping CI.

**Fig. 5:**
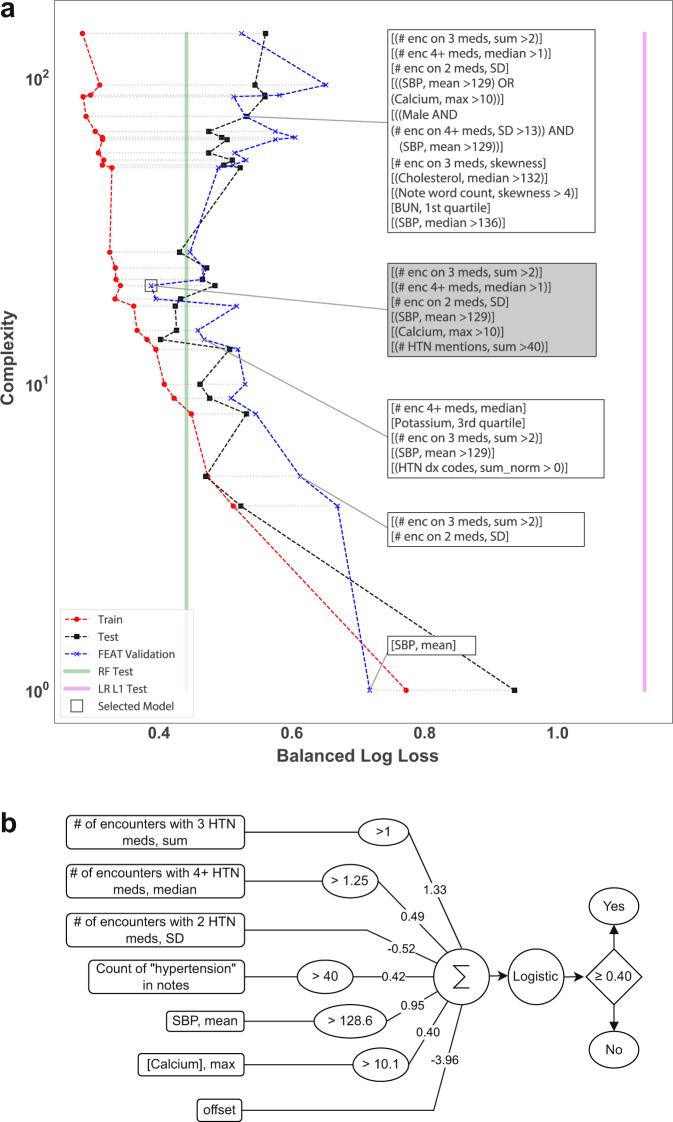
FEAT models trained to predict apparent treatment-resistant hypertension. **a** We plot the final Pareto archive of FEAT models for aTRH in terms of complexity versus balanced log-loss, with the latter shown for training, validation (used for model selection), and external test sets. The selected FEAT model is shaded gray. Vertical bars are shown for the test set performance of RF and LR L1 models. Note, for clarity of display the model numeric thresholds are rounded to the nearest integer (i.e. Calcium, max > 10.1 mg/dL as Calcium, max > 10). **b** Depiction of the selected FEAT model for classifying subjects as having apparent treatment-resistant hypertension. The input features are shown on the left followed by the learned transformation operations, the multiplication coefficients, the summation, logistic transformation, and decision threshold.

To provide further insight into the model construction process, we inspected the full Pareto-optimal set of models FEAT trained to predict aTRH by chart review (Fig. 5a). In the training set, as model complexity increased model performance similarly increased. However, as expected, in both the validation and held-out test data, there was an inflection point above which additional complexity was associated with poorer performance. The final model was selected as the one with the lowest balanced log-loss in the validation set. This model showed similar performance in the held-out testing set. Notably, the models adjacent to this inflection point demonstrated balanced log-loss comparable to that of the RF model and far superior to that of the LR L1 model.

### Assessment of model generalization and clinical utility

To further evaluate the utility of the resulting models, we selected diagnostic interpretive thresholds. For the clinical goal of identifying patients that should be screened for PA using models predicting aTRH, we targeted a model PPV for aTRH of ≥0.70 amongst primary care patients. We assumed an aTRH prevalence of 7.5%, based on the frequency observed in our training set and a meta-analysis^48^. Based on the estimate that 20% of aTRH patients have underlying PA, we expect that approximately 1 in 7 aTRH prediction model-positive patients would have PA. In training, we selected a classification threshold of 0.40, which corresponded to an observed sensitivity of 0.82. Among the 200 randomly drawn held-out test subjects, this FEAT model yielded an adjusted PPV of 0.70 and sensitivity of 0.62. To evaluate FEAT on a richer set of cases, we also assessed its performance on 100 test patients positive for the final aTRH or HTN-hk heuristics. In this set, the final FEAT model demonstrated a PPV of 0.79.

To further evaluate the generalization of FEAT-trained models to external data, we performed an internal-external validation study in which models were trained in subjects from 70% of primary care practice sites and evaluated in a held-out set of subjects from 30% of primary care practice sites. The FEAT-trained models showed very similar performance to when sites’ data were randomly spread over both training and testing, including an AUPRC for aTRH of 0.77 (95% CI: 0.63–0.89; Supplementary Table 5). Notably, while all methods showed lower AUPRC for HTN-hk by chart review in this evaluation, the FEAT model (AUPRC: 0.92 (CI: 0.85–0.96)) appeared to generalize better than several other methods, including the more complex RF model (AUPRC: 0.88 (CI: 0.80–0.95)).

### Model interpretability

We next evaluated the relative interpretability of the resulting models, focusing on the models for predicting aTRH. The final FEAT model (Fig. 5b) was concise and fully specifiable. This model assigned risk according to the following factors, in order of absolute coefficient magnitudes: first, a history of more than one encounter while prescribed three or more anti-hypertensive medications (β = 1.33); second, a mean systolic blood pressure above 128.6 mmHg (β = 0.95); third, a history of low variability (standard deviation) in the number of encounters while prescribed two anti-hypertensive medications each year (β = -0.52); fourth, a history of a median of 1.25 or more encounters per year while prescribed four or more hypertension medications (β = 0.49); fifth, more than 40 mentions of hypertension in clinical notes (β = 0.42); and sixth, a maximum total calcium greater than 10.1 mg/dL (β = 0.40). To investigate the factors underlying the maximum calcium feature, we explored its associations. We found that subjects with aTRH were in fact more likely to have an elevated maximum calcium (OR = 4.4; *p* = 4 × 10^−9^, Fisher’s Exact test) and that these elevations were in turn associated with the number of days prescribed thiazide diuretics (OR = 1.5 per SD; *p* = 3 × 10^−6^, *Z*-test) and beta-blockers (OR = 1.4; *p* = 2 × 10^−4^).

**Fig. 6:**
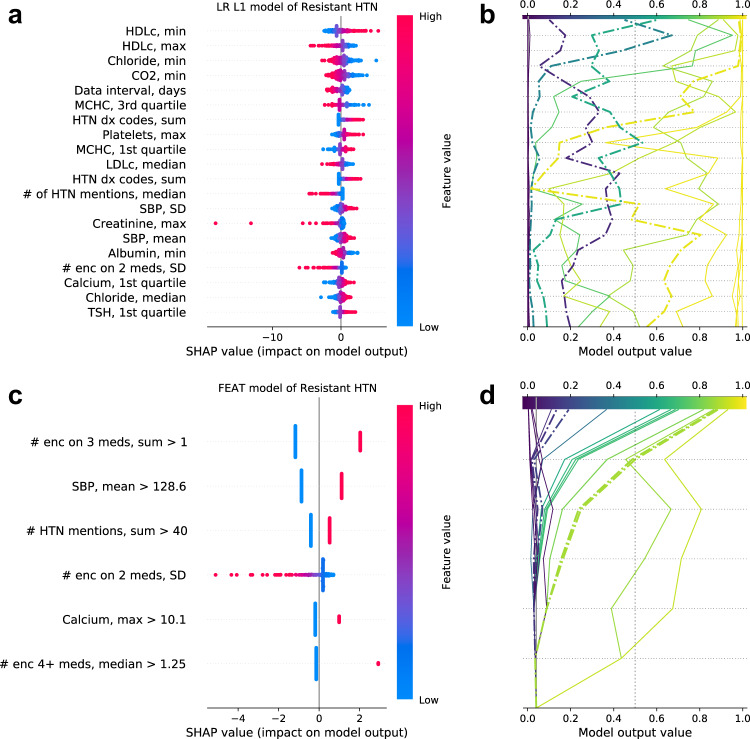
SHAP plots for explaining models. SHAP summary (**a**) and decision (**b**) plots for the LR L1 and summary (**c**) and decision (**d**) plots for the FEAT models. The summary plots (**a**, **c**) describe the most important features, ranked by the mean absolute SHAP value in the test data. Each point represents a subject; its color reflects the relative feature value and the location along x-axis its estimated impact on the subject’s model output. The lines in the decision plots (**b**, **d**) show model predictions for a sample of 10 positive and 10 negative predictions, with dash-dotted lines indicating misclassifications. The summary and decision plots are aligned vertically, such that the feature labels in the summary plots correspond to the incremental changes in the adjacent decision plot lines, indicating the feature responsible for the change in the model score at each level. Note, the x-axis for the decision plots (**b**, **d**) are restricted to 0–1.

None of the other methods’ trained models can be described in such compact and clear language. The other potentially interpretable modeling methods generated models that were too large to be understood at an intuitive level in practice. To directly compare and contrast the interpretability of FEAT and other methods, we calculated SHAP values^31^ for the test subjects. SHAP values summarize the impact of input variables on model outputs by generating an additive feature attribution model. Positive and negative SHAP values indicate a marginal increase and decrease in predictions, respectively. The summary plots for SHAP values (Fig. 6a, c) depict the distribution of SHAP values relative to the magnitude of each input variable, with each dot representing a single test subject. The decision plots for SHAP values (Fig. 6b, d) illustrate how each feature contributes to predictions for a subset of individual subjects.

The FEAT summary plot (Fig. 6c) reflects the simplicity of the FEAT model. For the five dichotomized features, each patient’s prediction is either increased or decreased by a fixed increment. The one continuous feature affects each patient distinctly, but has a clear directionality, i.e. high variability in the number of encounters per year on two anti-hypertensive medications decreases the prediction. These simple effects translate into intuitive interpretations for individual subjects as to *why* the model is calling them positive or negative (Fig. 6d). The positive increases in model output show that most patients predicted to be positive have had multiple encounters while prescribed three anti-hypertensive medications. They also had either an elevated mean systolic blood pressure and many mentions of hypertension in notes or multiple encounters per year while prescribed four or more anti-hypertensive medications.

In contrast, the LR L1 (Fig. 6a, b) and RF (Supplementary Fig. 4) summary and decision plots reflect much more complicated models, in which many features contribute to the prediction scores. The summary plots show the modest effect of each of the 20 displayed features, sorted by average absolute variable importance. The decision plots demonstrate that each patient has a distinct *reason* for a positive or negative prediction, determined by a combination of many features. There is slow decay in importance across ranked features. In addition, there is also considerable signal from the features not depicted, as evident in the variable, non-zero intercepts between each patient’s line and the model output value x-axis. Thus, one cannot simply identify specific factors that explain LR L1 or RF classifications. For example, there are many features that appear to have had small, positive impacts resulting in misclassification of the single depicted false-positive subject (Supplementary Fig 4B, dot-dashed line with model output probability greater than 0.5). The mechanism by which each feature contributes to the misclassification cannot be deduced without fully considering the interactions between features in the ensemble. In contrast, since FEAT performs logistic regression on the transformed features (Fig. 6), the derived predictors have linear and additive impacts on model output that can explain misclassifications.

Notably, for the LR L1 explanations in Fig. 6 many of the top features (e.g. minimum HDL cholesterol) are not intuitively linked to the phenotype, likely due to feature co-linearity. To address this, we also calculated LR L1 SHAP values after adjusting for feature covariance (Supplementary Fig. 5A, B). In this case, SHAP values do not explicitly represent linear model coefficients. Instead, SHAP values are transformed by applying a linear projection to the input data and model coefficients. Put simply, whereas Fig. 6 SHAP values are faithful to the models and its coefficients, Supplementary Fig. 5 SHAP values are more faithful to the correlation structure of the input data. After adjustment, the top features (e.g. # enc 4+ meds, median) more closely matched clinical intuition, including small positive effects on aTRH predictions from encounter counts while prescribed multiple medications, systolic blood pressure summarizations, and counts of days on hypertension medications. However, the relationships between features and SHAP values remained complex, including a large number of features with small individual effects. In addition, to identify the relationships between such features and the LR L1 model predictions requires a close inspection of the data, and is not apparent from simple inspection of the model coefficients themselves (i.e. Fig. 6). For the sake of comparison, we also accounted for co-linearity in the FEAT model (Supplementary Fig. 5C, D). While these FEAT model explanations do show some smearing of the features’ apparent impact, the overall interpretability and interpretation of the model does not fundamentally change.

### Method generalizability across common phenotypes in open, benchmark EHR tasks

Finally, to assess the generalizability of this approach to many important clinical use cases and other clinical data sources, we leveraged an existing data pipeline^23^ for the large, publicly available, and well-studied MIMIC-III critical care database^46^. We applied FEAT in comparison to LSTMs and LR on 25 benchmarking clinical phenotype prediction tasks. In contrast to the PA chart-reviewed phenotyping, these data consist mostly of time series, which LSTMs train on directly. We trained models using data from 35,621 patients and evaluated models for 6281 patients.

**Fig. 7:**
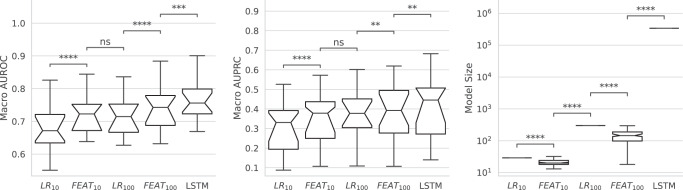
Performance across benchmark phenotyping tasks. We compare FEAT to penalized LR and deep neural networks (LSTM) on 25 established phenotyping tasks using the MIMIC-III critical care database. To compare FEAT and LR at similar levels of interpretability, we restrict the final model dimensionality to 10 or 100 features, as noted. Macro-averaged AUROC, AUPRC, and model size comparisons are shown. Boxplot notches indicate bootstrapped 95% confidence intervals (CIs) of the medians. Wilcoxon rank-sum pairwise significance comparisons are shown as follows: ns: *p* < = 1; *0.01 < *p* < = 0.05; **0.001 < *p* < = 0.01; ***1e–04 < *p* < = 0.001; ****p < = 1 × 10^−4^. Box centerline: median; box limits: quartiles; whiskers: 1.5x the interquartile range.

To explore the tradeoff between model performance and complexity, we compared two versions each for FEAT and LR that learn models with up to either 10 or 100 dimensions. Limiting models to 10 dimensions to maximize interpretation, FEAT models demonstrated discrimination (macro AUROC [mAUROC] = 0.72; macro AUPRC [mAUPRC] = 0.35) that outperformed LR (mAUROC = 0.68, *p* = 6.0 × 10^−6^; mAUPRC = 0.30, *p* = 3.4 × 10^−6^; Fig. 7, Supplementary Fig. 6 and Table 6). Notably, these 10-dimension FEAT models performed similarly to 100-feature LR models (mAUROC = 0.72, mAUPRC = 0.37; *p* = 1.0). Across tasks there was considerable variability in model discriminative performance (Supplementary Fig. 6). The LSTM models were 4 orders of magnitude larger and demonstrated slightly higher discrimination (mAUROC = 77, mAUPRC = 0.41); allowing FEAT to learn 100-dimensions enabled it to approach this discriminative performance (mAUROC = 0.74, mAUPRC = 0.38; *p* = 0.005).

## Discussion

We adapted and further developed a symbolic regression method FEAT for constructing EHR prediction models that are both accurate and intuitively interpretable and then applied it to find patients that should be screened for PA and to predict 25 phenotypes in established benchmark tasks. This approach yields clinically intuitive models by embedding the design goals from manual curation of phenotyping heuristics: high accuracy and low complexity. Importantly, FEAT achieves this in a largely automated process such that, unlike manual curation, it could be applied at scale for a wide expanse of clinical use cases across many clinical practices’ data.

There is an important and active debate regarding the importance of model interpretability in achieving acceptance, trust, and effectiveness in ML-triggered CDS^16,17^. Much of that debate considers the relative utility of post-hoc explanations of black-box models because of the performance limitations of conventional interpretable modeling methods. We demonstrate that for many important clinical use cases FEAT can learn models that are concise enough to be inherently interpretable, circumventing the challenges and limitations of trying to accurately and precisely explain a model’s predictions post-hoc. This level of understanding of model predictions should contribute to safer and more effective ML-triggered CDS and be advantageous in an important set of clinical use cases for which interpretable model performance rivals that of black box methods or a modest cost in performance is outweighed by the benefits conferred by interpretability in improved trust and understanding.

We compared the ability of FEAT to learn accurate and interpretable prediction models from EHR data to that of conventional ML methods. The models FEAT learned to predict the PA screening chart-review phenotypes were more concise (CV range: 1.7-fold to 4000-fold; *p* < 0.001) and more interpretable (Figs. 5b & 6) than those of all other ML approaches. In addition, their discriminative performance matched or exceeded that of all other potentially interpretable ML methods (ΔCV AUPRC: +0.00 to +0.44). Similarly, FEAT trained concise and accurate models across 25 established phenotyping tasks using MIMIC-III data. We also compared FEAT to complex, black-box methods, demonstrating that FEAT models can achieve similar discriminative performance (FEAT-100 mAUPRC = 0.38 versus LSTM mAUPRC = 0.41). Although some of the FEAT models for PA prediction showed slightly lower AUPRC than corresponding RF models (e.g. CV AUPRC 0.69 versus 0.75 for aTRH chart review), the two methods generated similar balanced log-loss scores (the cost function optimized as part of the FEAT algorithm), as shown in Fig. 5a.

The model that FEAT learned to identify patients with aTRH was both accurate and understandable. FEAT learned to combine complementary sources of information, including medication, vitals, laboratory results, and concepts from notes. The model’s components were very similar to those of the expert heuristic and they matched factors expert clinicians use in evaluating for resistant hypertension, including the number of anti-hypertension medications, the duration and variability of these medication prescriptions, and blood pressure measurements. In addition, FEAT learned an unexpected but clinically intuitive and valuable rule related to maximum blood calcium levels. Anti-hypertensive medications, particularly diuretics, can dysregulate calcium homeostasis. In addition, hyperparathyroidism, which causes elevated blood calcium, is associated with hypertension. We suspect this rule enabled the model to identify a few affected subjects, either on intensive anti-hypertensive regimens or with underlying hyperparathyroidism, who were missed by expert-curated heuristics that only consider medication prescriptions and blood pressure.

In applying FEAT to identify patients that meet the two major clinical guideline criteria for PA screening, aTRH and HTN-hk, we constructed prediction models that could be used to trigger decision support for PA screening. Expert-curated computable phenotypes have been constructed for PA screening criteria, including for aTRH^49,50^. But to our knowledge this is the first demonstration of a ML-trained phenotyping model for this purpose. Critically, the FEAT-trained model achieves our clinically derived goals for (1) specificity in our target population (PPV ≥ 0.7) such that we would expect >10% of model-positive patients to have undiagnosed PA, and (2) interpretability (Fig. 5b). The FEAT-trained models are preferable to those trained by conventional interpretable ML modeling methods because of their comparable or superior discrimination (ΔCV AUPRC: +0.0 to +0.44) and better interpretability (Fig. 5b & 6). The FEAT models are also preferable to the RF models in this case, because the small decrement in discrimination (e.g. CV AUPRCs 0.69 versus 0.75 for aTRH chart review) is outweighed by the considerable improvement in interpretability (Figs. 5b & 6c, Supplementary Fig. 4) and thereby likely better compatibility with corroborating or second-guessing model predictions as part of conventional clinical decision making practices.

We note several study limitations. First, the FEAT models we have trained would require additional refinement and validation prior to implementation in clinical practice. As this work’s goal was to present and evaluate the method and approach rather than to build a specific, implementable model, we did not assess the generalizability of specific FEAT-trained models across healthcare systems. However, to assess the approach’s potential for learning generalizable models, we did perform an internal-external validation study in which models were trained and tested in patients from separate primary care practices. Although there is likely more variability between health systems than between clinical practices within a health system, this validation study demonstrated the generalizability of FEAT-trained models to held-out clinical practices (Supplementary Table 5). For aTRH by chart review, FEAT achieved a test AUPRC of 0.77, which was similar to that of both the LR L1 (AUPRC = 0.78) and FEAT in the non-site-randomized, held-out testing (AUPRC = 0.78) models. This cross-site generalizability is expected because FEAT uses an internal held-out validation set to select a final model from a set of models trained in parallel (see Fig. 5a) and because the lower complexity of FEAT models should decrease overfitting. In fact, FEAT models appeared to generalize better than some other models, including RF models that showed AUPRCs that were lower by 0.08 (HTN-hypoK) and 0.05 (aTRH) in subjects from held-out practice sites.

A second study limitation is that we only compared FEAT to a select number of other ML methods. In our PA studies as our primary focus was on interpretable models, we chose to only compare to a single ‘black-box’ method, RF. In our follow-up MIMIC-III studies that leveraged time series data, we compared to a single deep learning method (LSTM). In each of these studies, we could have included additional methods that may have demonstrated slightly better classification than those we reported. However, we believe this would not meaningfully change the interpretation of this work because (a) we have previously compared FEAT to a large variety of methods and (b) none of the conventional methods expected to yield slightly higher accuracy would be comparably interpretable^32,33^. In addition, as the relative performance of these methods depends on the use case, adding these analyses here would not change our recommendation that when developing ML models for CDS one should directly evaluate the relative discriminative performance of FEAT and other potential methods for each specific clinical use case.

A third related study limitation is the comparator methods likely could have been further refined to achieve better interpretability and accuracy. For instance, while in our MIMIC-III analyses we performed feature selection prior to LR in order to compare FEAT and LR classification performance at similar model complexity, we did not do this in the PA studies. We felt this comparison was unnecessary as in the PA studies the much more complex LR models did not demonstrate superior classification. We expect that if we were to decrease the LR models to the size of the FEAT models, the smaller LR models’ discriminative performance would be lower than that of the FEAT models. Similarly, we could have explored other options for further optimizing comparator method hyperparameters. However, the implementations we applied were consistent with conventional best practices and we would not expect further refinement to dramatically affect comparator method performance.

We also note a few limitations to the presented FEAT method. First, the FEAT models showed lower discriminative performance than ‘black box’ modeling methods for some complex tasks. To train models that are concise and interpretable, we restricted the operator space and the size of learned models. This emphasis on small size was necessary to achieve interpretability, but it came at a cost to discriminative performance for more complex tasks. For examples, for aTRH some patients with heart failure or chronic kidney disease were misclassified by FEAT as positive (Fig. 6d), but the larger LR L1 model lowered prediction scores based on associated clinical features maximum creatinine or heart failure diagnosis codes (Supplementary Fig. 5A). Related features were observed in FEAT models along the Pareto-optimal front during training (Fig. 5b and data not shown), but these models were ultimately not selected due to their higher overall validation loss. Such models may have been selected were more training cases available. Across the 25 MIMIC-III phenotyping tasks, the magnitude of the tradeoff between interpretability and discrimination varied. For several tasks, in order to approach the discriminative performance of LSTM, FEAT models’ dimensionality needed to be less constrained so that it could learn more discriminative models. These larger models are unlikely to be intuitively interpretable in the same way as a 10-dimensional model can be. With this in mind, FEAT will not be the best tool for every use case. For a given clinical use case, the utility of FEAT compared to other methods will depend on the observed tradeoff between of interpretability and discriminative performance and use-case specific factors, including implementation contextual factors such as the proposed role of the model in clinical decision making and the cost (including financial cost, patient and clinician time, and potential for harm) of the next step in the clinical workflow. That said, we expect the tradeoff between discrimination and interpretability could be improved by further method development. Since the balanced log-loss, which is the metric FEAT was optimizing, was similar between the FEAT and RF models, we plan to explore more effective cost function proxies for model target performance metrics (e.g. AUPRC).

A third limitation, which if addressed by future work could likely improve the performance of FEAT models, is the reliance on pre-processing of input clinical data upstream of FEAT. This pre-processing selects and compiles data elements from across large databases consisting of many separate tables and transforms longitudinal features into individual, summary patient-level features. It is very important to consider this pre-processing as both the interpretability and accuracy of the end-model is dependent on it. However, this deficit is not specific to FEAT, as comparable pre-processing is also required for conventional potentially interpretable methods and some ‘black box’ methods (e.g. RF). We applied two distinct approaches to feature engineering. For PA, we leveraged expert knowledge to derive some use case-specific input features, such as “# of encounters with 3 HTN meds, sum”. In contrast, for MIMIC-III in order to scale to many more phenotypes, we applied an automated approach that was not informed by external expert knowledge. We expect that if we had leveraged external knowledge in the MIMIC-III analyses, the FEAT model discriminative performance could have been improved for some phenotypes. To scale this approach to effectively leverage external knowledge in an automated way for new use cases, further methodological development would be needed. External knowledge could be incorporated by leveraging clinical vocabularies and ontologies for representing both clinical data and expert clinical knowledge^51,52^. For example, the importance of counting the number of prescribed HTN meds could be learned by aggregating medication prescriptions using grouper variables defined in EHRs or standard vocabularies (e.g. RxNorm). In addition, if the method were able to directly operate on longitudinal data (like LSTMs) it could learn cross-feature longitudinal interactions such as “elevated BP *while* prescribed 3 + HTN medications”.

A fourth limitation of the presented method is that the FEAT models are not guaranteed to be as clinically intuitive as expert curated heuristics. Our modifications to FEAT successfully constrained the size of models and the operators used in models, which is a necessary component for interpretability. However, these constraints do not guarantee that the models are also intuitive. Intuitive interpretability places further requirements on the processing of input data and the model component operations. One future opportunity for improving interpretability is building on top of clinical concepts from ontologies, as described above. Another direction for improving interpretability is further simplifying the model operations. For instance, simplifying threshold choices so that instead of learning a BP threshold of 128.6 mmHg and Calcium threshold of 10.1 mg/dL, FEAT would consider less precise but more intuitive thresholds of SBP ≥ 130 mmHg, Calcium ≥ 10 mg/dL, or high Calcium (i.e. Calcium above the upper limit of the reported reference interval).

In summary, FEAT can effectively learn EHR prediction models that are both highly accurate and highly interpretable. The FEAT-trained PA screening models could be implemented, following further refinement and validation, to trigger decision support safely and accurately. We expect that this modeling approach will ultimately enable scaling of the learning of safe, effective ML models across clinical use cases and healthcare practices, facilitating widespread implementation of targeted, efficient CDS.

## Methods

### Feature engineering automation tool (FEAT)

We extended a recent method for learning informative feature representations called FEAT (v0.4.2) to improve its ability to construct interpretable EHR prediction models (Fig. 1)^32–34^. Our goal was to build a classification model from a set of *N* paired samples, $\left\{\left({y}_{i},{{\boldsymbol{x}}}_{i}\right),{\rm{i}}=1,\ldots ,{\rm{N}}\right\}$, with binary labels $y\in \{\mathrm{0,1}\}$ and attributes ${\boldsymbol{x}}\in {{\boldsymbol{R}}}^{d}$. FEAT attempts to learn a set of features for a logistic model of the form

1 $$\begin{array}{c}{logit}\left(P\left(y=1|{\boldsymbol{x}}\right)\right)={{\boldsymbol{\beta }}}^{T}{\boldsymbol{\Phi }}\left({\boldsymbol{x}}\right)\end{array}$$

where ${\boldsymbol{\phi }}\left({\boldsymbol{x}}\right)$ is a feature representation, i.e., a *p*-dimensional vector of transformations of ${\boldsymbol{x}}$, and ${\boldsymbol{\beta }}=\left[{{\rm{\beta }}}_{1},\ldots ,{{\rm{\beta }}}_{p}\right]$ is the associated vector of coefficients. Like other symbolic regression methods^53^, FEAT generates candidate representations ${\boldsymbol{\phi }}\left({\boldsymbol{x}}\right)$ by searching a space of expression trees composed of simple functions.

The goal of FEAT is to estimate the form of ${\boldsymbol{\phi }}\left({\boldsymbol{x}}\right)$ and the ${\boldsymbol{\beta }}$ coefficients that minimize two quantities: (1) the balanced logistic loss of the ${{\boldsymbol{\beta }}}^{T}{\boldsymbol{\Phi }}({\boldsymbol{x}})$ prediction in the training set, and (2) the complexity of ${\boldsymbol{\Phi }}({\boldsymbol{x}})$ (Fig. 1b). In brief, FEAT accomplishes this using a genetic programming approach in which a set of candidate representations iteratively undergo selection, variation, and survival operations (Fig. 1a). The selection step applies epsilon-lexicase selection^54^, which helps preserve representations that perform well for rare and/or difficult subjects. The variation step applies insertion, deletion, point mutation, and crossover functions to selected representations. The survival step uses a variant of the multi-objective optimization algorithm NSGA-II^55^ for which the main driving concept driving optimization is *Pareto dominance*: one candidate representation dominates another if it is at least as good in one of the two objectives (e.g., lower complexity) while being better in the other (e.g., lower logistic loss; Fig. 1b). The optimization procedure utilizes this concept to find a set of models that are non-dominated, meaning they are efficient trade-offs between complexity and error.

For model training, FEAT internally splits input data into training (80%) and validation (20%) sets. Model representations and coefficients are learned in the training set. Pareto-optimal models are then evaluated in the validation set and the model with the lowest balanced log-loss is selected. Classifier thresholds were selected in the combined training and validation set to achieve a positive-predictive value (PPV) in the longitudinal, primary care cohort of 0.70.

### FEAT methodological development

We made a series of methodological changes to FEAT to improve its ability to construct interpretable EHR prediction models. First, in order to generate models that are interpretable, we restricted the operators in these expression trees to Boolean functions {<, >, AND, OR, NOT}. Second, in contrast to traditional symbolic regression, we implemented inequality operators that learn splitting thresholds on input features based on Gini impurity, in a similar way to classification and regression trees (CART)^56^. Unlike decision tree algorithms, FEAT’s optimization process is non-greedy, allowing for globally optimal thresholds to be sampled.

To further encourage model parsimony, we refined FEAT by introducing two additional modifications to the FEAT algorithm (Supplementary Table 1) and one modification to our application of FEAT. These changes incorporated modifications (a) to sample features based on univariate logistic regression coefficients; (b) several modifications to shrink the feature representations without significantly altering its behavior, including pruning highly correlated feature branches, explicitly simplifying serial logical operators, and adaptively pruning components of representations; and (c) robust model selection favoring smaller models. The following sections describe these in more detail.

First, we altered the initial feature weighting. The original FEAT algorithm initialized weights of input features according to the magnitude of their coefficient in a multivariate linear model^33^. In addition, the initial population was seeded with the multivariate linear model that was generated. Since we are interested in learning a low-dimensional representation of high-dimensional data to enable interpretation, this approach was not suitable. Instead, we modified FEAT to specify initial weights of input features according to the magnitude of each feature’s coefficient in univariate logistic regression models. The initial population of linear models was constructed by sampling features according to these magnitudes and fitting a low-dimensional multivariate model.

Second, we added functionality to prune highly correlated feature branches. In previous work, operators for variation were introduced to make use of information about the features encoded by the representations^32^. Here, we propose an operator designed to prune representations by removing the most redundant feature (See Algorithm 1 in the Supplementary Notes). In short, it consists of computing pairwise correlations between all features, and among the pair that is most correlated, deleting the feature that is less correlated with the outcome variable.

Third, we added methods to explicitly simplify models. Genetic programming suffers from a phenomenon known as *bloat*, in which final equations that are produced tend to be larger than necessary for capturing their semantics^57^. Many methods exist to combat bloat^58,59^, including various pruning mutations such as Algorithm 1. A simple but effective way to reduce bloat is post-run simplification^60^, in which simplification operations are applied to the final model in a hill climbing manner. In order to avoid over-fitting, changes are only accepted if their cumulative effect on the model output is on average within a user-specified tolerance.

We introduced an automated method for simplifying final representations produced by FEAT that includes three steps. First, redundant operations, such as NOT(NOT(.)), are removed. Second, correlation deletion mutation is applied iteratively. Finally, a uniform subtree deletion operator is applied iteratively. Each iteration succeeds only if the impact on the final model performance is minimal, or, in the case of correlation deletions, if the features were perfectly correlated. Post-run simplification is shown concretely in Algorithm 2 in the Supplementary Information.

Finally, we applied an approach for robust model selection that favored smaller models. Due to its nature as a population-based method, FEAT’s optimization process produces several candidate final models along the Pareto-optimal front. In order to choose a single final model, models are trained on 80% of available training samples and 20% of training samples are held-out for internal model validation. Then from the population of models along the Pareto front, the model with the lowest balanced log-loss in the validation (held-out 20%) samples is selected as the final model. Due to its nature as a probabilistic algorithm, FEAT is sensitive to the random seed used in training. In order to encourage the selection of a robust final model, we designed a heuristic procedure. FEAT was rerun 10 times in training, thereby yielding 10 models. Of these final models, we excluded those in the lowest quartile of validation AUPRC and then chose the smallest model. In our preliminary cross-validation analyses, we found this to result in relatively stable, discriminative, and interpretable models over 50 realizations of our experiment. However, this procedure is ad hoc and a better approach may exist.

### Benchmarking variants of FEAT

Supplementary Table 1 describes 5 variants of FEAT that we benchmarked in order to validate the algorithmic changes proposed above. We conducted this experiment to test the following hypotheses: (1) restricting FEAT to boolean operators would produce simpler models; (2) the post-run simplification operator would produce simpler models; (3) post-run simplification would produce models with derived features that were more orthogonal; (4) the multi-dimensional architecture FEAT uses would perform better than an even simpler “single model” approach frequently used in genetic programming.

In order to test these changes generally, we chose a set of 20 benchmark classification problems from the Penn ML Benchmark (PMLB)^47^. These datasets are widely available, real-world and simulated problems. We chose 20 datasets whose shape (number of samples and features) was closest to that of the hypertension problems (Supplementary Table 2). For the PMLB comparisons, we ran 10 trials of shuffled 75/25 train/test splits.

### Method performance assessment

FEAT models were compared to conventional supervised classifiers from Scikit-learn^61^. Hyperparameters for each of the methods were optimized using 5-fold nested cross-validation. Specifically, random forest hyperparameters n_estimators (from = 100, to = 2100, num = 6) and max_depth (from = 100, to = 110, num = 6) were optimized in 5-fold inner cross-validation. Decision tree hyperparameters considered were max_depth (from = 10, to = 110, num = 11), min_samples_split (2, 5, or 10), and min_samples_leaf (1, 2, or 4). Logistic regression L1 and L2 methods penalties were optimized across 10 orders of magnitude from 10^−6^ to 10^−3^. For LSTM architecture, see section ‘MIMIC-III clinical data analyses’ below.

We first evaluated methods in 50 trials of 5-fold cross-validation on shuffled training datasets and averaged test scores across folds. We report the mean test AUPRC and AUROC for all experiments. AUPRC is calculated as average precision (see *average_precision_score* in scikit-learn version 0.23.2). We measured the size of the models for tree-based methods (FEAT, DT, and RF) as the total number of split nodes and leaf nodes in the trees. For the linear methods and GNB, in the PA studies the size is the number of predictors with non-zero coefficients. For the MIMIC-III benchmarks, we used a more complete measure of size for linear models that included multiplication operators and variables in the calculation. Models’ metrics were quantitatively compared using pairwise Wilcoxon rank-sum tests. Confidence intervals were estimated using 1000 bootstrap resamples. Study code, including full environment specification, is available in the repository https://bitbucket.org/hermanlab/ehr_feat/.

### PA patients

We studied 1200 patients receiving longitudinal primary care in the University of Pennsylvania Healthcare System (UPHS). Subjects included had (a) at least five outpatient visits in at least three separate years between 2007 and 2017, (b) at least two encounters at one of 40 primary care practice sites, and (c) were 18 years or older in 2018. A set of 1000 random subjects from this cohort were divided into 800 for model training (and validation) and 200 for model testing. One subject in the random training set was excluded because of a mid-study change in enterprise master patient index (EMPI) identifier.

Preliminary and final expert-curated heuristics for aTRH and HTN-hk were used to identify an additional 50 subjects each for model training and model testing. This yielded a total of 899 subjects for the training set and 300 subjects in the testing set.

This study followed all relevant ethical regulations. The protocol was reviewed and approved by the University of Pennsylvania Institutional Review Board (#827260), which approved a waiver of informed consent.

### Clinical chart review

A study physician reviewed clinical charts and classified subjects with respect to three phenotypes of increasing complexity for hypertension related to screening guidelines for PA: hypertension, hypertension with unexplained hypokalemia (HTN-hk), and apparent treatment-resistant hypertension (aTRH). The chart review process and form was designed and reviewed by three study physicians (I.A., D.S.H., and J.C.). Classification was based on JNC7 Guidelines on Prevention, Detection, Evaluation, and Treatment of High Blood Pressure^62^. Chart review was primarily performed by one clinician reviewer (I.A) who was a MD graduate with practice experience as a medical officer. Unclear cases were reviewed by one additional study physician (either D.S.H. or J.C.). J.C. is a hypertension specialist.

Chart review results were documented in a single Redcap form, including the reviewer’s summative conclusion (i.e. yes/no for resistant hypertension) and the underlying evidence (e.g. # of concurrent anti-hypertension medications, # of elevated blood pressure measurements, alternative explanations for elevated blood pressure such as acute illness, and exclusion criteria).

Patients were deemed to have hypertension if they had multiple documented elevated blood pressure measurements (SBP ≥140 mmHg or DBP ≥ 90), were being treated with an anti-hypertensive medication for blood pressure control, or had documented hypertension in diagnosis codes or notes. Elevated blood pressures were considered not indicative of hypertension if there was no clinical diagnosis and the elevation was potentially explained by clinical context, such as acute illness or pain, or interpreted as situational (e.g. white coat hypertension) and not treated as hypertension.

Patients were considered to have hypokalemia if there was documented evidence of an outpatient laboratory test result with low potassium or were prescribed outpatient oral potassium supplementation. Hypokalemia was considered *explained* if the measurements coincided with a dilutional explanation (e.g. saline infusion, chemo-infusion), acute illness potentially explaining (e.g. gastroenteritis with vomiting and diarrhea), dietary restriction, medication with known side effect (e.g. Bortezomib, amphotericin B), or hypomagnesemia.

Patients were considered to have apparent treatment-resistant hypertension (aTRH) if they were on anti-hypertension medications from 4 distinct classes for at least a month or from 3 distinct classes for over a month and had multiple elevated blood pressure measurements that did not appear to be explained by identifiable factors (e.g. medication adherence, insufficient dosing, acute illness). Patients with evidence of heart failure or chronic kidney disease prior to meeting aTRH criteria were considered negative.

### PA clinical data

We extracted 331 features from the EHR clinical data repository Penn Data Store and EPIC Clarity reporting database (Supplementary Tables 7–12). Demographic and encounter features included age, race, sex, categorized distance from zip code 19104, weight, BMI, blood pressures, and number of elevated blood pressures. Longitudinal features were aggregated as minimum, maximum, median, standard deviation, and skewness. The 34 most common laboratory test results (complete metabolic panel, complete blood count with differential, lipids, thyroid stimulating hormone, and hemoglobin A1c) with <33% missingness were summarized as minimum, maximum, median, 1st quartile, and 3rd quartile. Diagnosis codes for hypertension, associated comorbidities, and other indications for anti-hypertensive medications were aggregated and summarized as median per year and sum. Medication prescriptions were summarized as the number of days prescribed for each antihypertensive class and the counts of encounters while prescribed 1, 2, 3, or 4 or more anti-hypertensive medications, summarized as sum, median, standard deviation, and skewness, as well as the sum of encounters with elevated blood pressures. Regular expressions, adapted with modifications from Teixeira et al.^63^, were applied to clinical notes to identify mentions of ‘hypertension’ and variants thereof, summarized as counts. Features with values outside of physiologically reasonable ranges, with fewer than 5% non-zero counts, or with variance below than 0.05 were excluded. Missing values were median imputed.

### Construction of expert-curated heuristics

To provide fully specified and clinically relevant outcomes for evaluating ML methods, heuristics were manually curated for the three target phenotypes by expert review of EHR data. This involved several iterations of proposing, applying, and evaluating the heuristics (led by D.S.H, I.A., X.D. and supported by J.C.). Heuristics were initially developed based on clinical and clinical data expertise and iteratively refined based on evaluation in serial sets of random training subjects. A preliminary set of heuristics for HTN-hk and aTRH were used to identify 50 patients, which were then used to further refine the criteria. Thus, final heuristics were developed from the entire set of 799 random and 100 targeted training subjects. Final heuristics were subsequently used to identify an additional 100 subjects for the held-out testing set.

The heuristic designed for hypertension queried for a history of two or more diagnosis codes for hypertension (International Classification of Diseases [ICD]-9: 401.*, 405.*; ICD-10: I10.*, I15.*). For HTN-hk, we labeled patients with at least two diagnosis codes for hypokalemia (ICD-9: 276.8; ICD-10: E87.6), or at least two outpatient encounters with low blood potassium (<3.6 mmol/L), or at least two prescriptions for an oral potassium supplement. For aTRH, we revised a previously reported heuristic^64^ to label patients (1) with documentation of at least 2 out of 5 consecutive outpatient encounters with elevated blood pressure (systolic blood pressure ≥140 mmHg or diastolic blood pressure ≥90 mmHg) while on antihypertensive medications from 3 distinct classes for at least 30 days prior to the elevated blood pressures or (2) prescribed four or more antihypertensive drug classes for at least 30 days. Exclusion criteria for aTRH included patients with a diagnosis code for heart failure or transplant (ICD-9: 428.*, V42.1; ICD-10: 150.*, Z94.1) or moderate to severe chronic kidney disease (estimated glomerular filtration rate [Modification of Diet in Renal Disease; MDRD]) <45 mL/min/1.73 m^2^ prior to meeting the above criteria.

### Association between laboratory results and medications

To understand the maximum calcium feature that FEAT learned to classify apparent treatment-resistant hypertension, we performed multivariate logistic regression considering all anti-hypertensive medication features using backwards selection, optimizing for Bayesian Information Content. Univariate relationships were evaluated using Fisher’s Exact tests and associations in multivariate logistic regression models were evaluated using *Z*-tests.

### MIMIC-III clinical data analyses

We constructed clinical phenotyping benchmark tasks from MIMIC-III data using the data pipeline provided by Harutyunyan et al.^23^ for extracting subjects, removing outliers, validating events, extracting episodes from subjects, and producing training, validation, and test sets. The dataset consisted of longitudinal measures for 17 clinical variables, including observations such as Glasgow coma scale, heart rate, oxygen saturation, temperature, weight, pH, and others (See Table 3 in Harutyunyan et al. for a complete list)^23^ from 42,276 ICU stays. For LR and FEAT models, we apply a standard time series feature extraction tool known as tsfresh^65^ that automatically extracts time series features and filters them on the training set using hypothesis tests incorporating training outcomes. Using tsfresh resulted in a large number of predictors, ranging from 1073 to 5976 across the phenotypes.

Given the large dimensionality of these data, to compare between FEAT and LR models we similarly restricted each to a specific numbers of dimensions: 10 and 100. We did not attempt to restrict the complexity of the LSTM. We used the LSTM architecture from previous work, consisting of 256-dimension LSTM layer with dropout set to 0.3, trained for 100 epochs with a batch size of 8. A 20% validation set was used to select the best epoch. An identical validation set was used by FEAT to select the final model from the Pareto archive for each task. For LR, this validation set was used to select the final hyperparameters for the model for each task. The hyperparameters included the type of regularization norm (L1 or L2) and the strength of this regularization in powers of ten.

### Reporting summary

Further information on research design is available in the Nature Research Reporting Summary linked to this article.

## Supplementary information

Supplementary Material

Reporting Summary

## Data Availability

The PMLB data is available from https://github.com/EpistasisLab/pmlb. MIMIC-III data is available from https://physionet.org/content/mimiciii/1.4/. The other EHR data in this article cannot be shared publicly to protect the privacy of the subjects. However, upon request and subject to appropriate approvals, it will be shared by the corresponding author.

## Code availability

FEAT is available from https://github.com/cavalab/feat; v0.4.2 was used in this paper. Study code is available in the repository https://bitbucket.org/hermanlab/ehr_feat/.
