# Supplementary material for: A flexible symbolic regression method for constructing interpretable clinical prediction models
Source: NPJ Digit Med. 2023 Jun 5;6:107. doi: 10.1038/s41746-023-00833-8 (PMC10241925; doi:10.1038/s41746-023-00833-8)
Supplement: Supplementary file 1 — Supplementary Material [file 41746_2023_833_MOESM1_ESM.pdf]

<u>Supplementary Information:</u> A flexible symbolic regression method for constructing interpretable clinical prediction models

William G. La Cava[1]**, Paul C Lee[2]**, Imran Ajmal[2], Xiruo Ding[2], Priyanka Solanki[2], Jordana B Cohen[3,4], Jason H Moore[4], Daniel S Herman[2]*

*[1]Computational Health Informatics Program, Boston Children's Hospital, Harvard Medical School, Boston, USA; [2]Department of Pathology and Laboratory Medicine, [3]Division of Renal-Electrolyte and Hypertension, Department of Medicine, and [4]Department of Biostatistics, Epidemiology, and Informatics, University of Pennsylvania, Philadelphia, USA. **equal contribution; *corresponding author.*

Supplementary Notes

Interpreting Prediction Models

There are two overarching approaches to explainable ML. The first is to apply a post-hoc analysis tool to a black-box model that determines which factors are relevant to the model's predictions.[1] Examples of post-hoc methods include permutation importance[2,3], LIME[4], and SHAP[5]. SHAP values in particular can be very useful for describing how a black-box model behaves under specific input conditions.[6] However, these approaches do not describe the *mechanism* by which factors result in the predictions. Furthermore, since these tools cannot describe the behavior of the model over all input conditions, it is challenging to predict model behavior on unseen inputs, especially when the model is highly non-linear.[7]

The second approach to explainable modeling is to focus on learning concise models that are instead self-explanatory. As Lundberg et al. put it, "the best explanation of a simple model is the model itself."[5] The most commonly used classification method in this category is logistic regression, often employed with regularization approaches, such as the least absolute shrinkage and selection operator (LASSO) and ridge regression.[8,9] Decision trees and Bayesian rule lists can also generate

interpretable models when constrained to small tree depths and low rule count, respectively. Yet these approaches are limited in that smaller models may not adequately represent complex data relationships and larger models are not practically interpretable.[10] In regularized regression and pruned decision trees, the trade-off between simplicity and explanatory power is left to be tuned by the user. More sophisticated strategies can characterize the trade-off between model complexity and model accuracy, such as Pareto optimization with symbolic regression.[11] *Symbolic regression* is a method of learning the functional form and parameters of a model using a randomized, heuristic search process such as evolutionary computation.[12] *Pareto optimization* refers to a multi-objective optimization process in which preference relations between models are determined by their closeness to the “Pareto front”, which is a set of points that represent the best observed trade-offs between objectives. Symbolic regression with Pareto optimization has been used to develop simple models in other domains, such as physics,[13] biology,[14] engineering.[15] To our knowledge, this is the first work to explore the application of symbolic regression with Pareto optimization to EHR phenotyping.

Algorithms

Algorithm 1: Correlation Deletion Mutation

```
CorrelationDeletionMutation($\hat{\boldsymbol{y}}(\boldsymbol{\Phi}(\boldsymbol{x}))$):
for $\boldsymbol{\phi}_i$, $\boldsymbol{\phi}_j$ in $\boldsymbol{\Phi}(\boldsymbol{x})$, $\boldsymbol{i} \neq \boldsymbol{j}$:
        corr = $\boldsymbol{R}^2(\boldsymbol{\phi}_i, \boldsymbol{\phi}_j)$
        max_r2 = 0.0
        if corr > max_r2:
                max_r2 = corr
                f1 = i
                f2 = j
corr_f1 = $\boldsymbol{R}^2(\boldsymbol{\phi}_i, \boldsymbol{y})$
corr_f2 = $\boldsymbol{R}^2(\boldsymbol{\phi}_j, \boldsymbol{y})$
Remove $\boldsymbol{\phi}$ from $\boldsymbol{\Phi}(\boldsymbol{x})$ with lower corr with $\boldsymbol{y}$
RETURN ($\hat{\boldsymbol{y}}_{\boldsymbol{new}}(\boldsymbol{\Phi}(\boldsymbol{x}))$, max_r2)
```

Algorithm 2: Post-run Simplification

```
PostRunSimplification( $\hat{\boldsymbol{y}}(\boldsymbol{\Phi}(\boldsymbol{x}))$, tol):
    $\hat{\boldsymbol{y}}(\boldsymbol{\Phi}(\boldsymbol{x}))$ - final model
    tol - tolerance for changes to output
    $\hat{\boldsymbol{y}}_{\boldsymbol{new}}$ = RemoveRedundantOperators($\hat{\boldsymbol{y}}$)
    for $|\boldsymbol{\Phi}(\boldsymbol{x})|$ iterations i:
            $\hat{\boldsymbol{y}}_{\boldsymbol{tmp}}$, max_r2 = CorrelationDeletionMutation($\hat{\boldsymbol{y}}_{\boldsymbol{new}}$)
            if ($\|\hat{\boldsymbol{y}}_{\boldsymbol{tmp}} - \hat{\boldsymbol{y}}\| / \|\hat{\boldsymbol{y}}\| <$ tol OR max_r2 == $\mathbf{1}$):
                    $\hat{\boldsymbol{y}}_{\boldsymbol{new}} = \hat{\boldsymbol{y}}_{\boldsymbol{tmp}}$
            else: break
    for 1000 iterations:
            $\hat{\boldsymbol{y}}_{\boldsymbol{tmp}}$= SubtreeDeletionMutation($\hat{\boldsymbol{y}}_{\boldsymbol{new}}$)
            if ($\|\hat{\boldsymbol{y}}_{\boldsymbol{tmp}} - \hat{\boldsymbol{y}}\| / \|\hat{\boldsymbol{y}}\| <$ tol):
                    $\hat{\boldsymbol{y}}_{\boldsymbol{new}} = \hat{\boldsymbol{y}}_{\boldsymbol{tmp}}$
    RETURN $\hat{\boldsymbol{y}}_{\boldsymbol{new}}(\boldsymbol{\Phi}(\boldsymbol{x}))$
```

Supplementary Figures

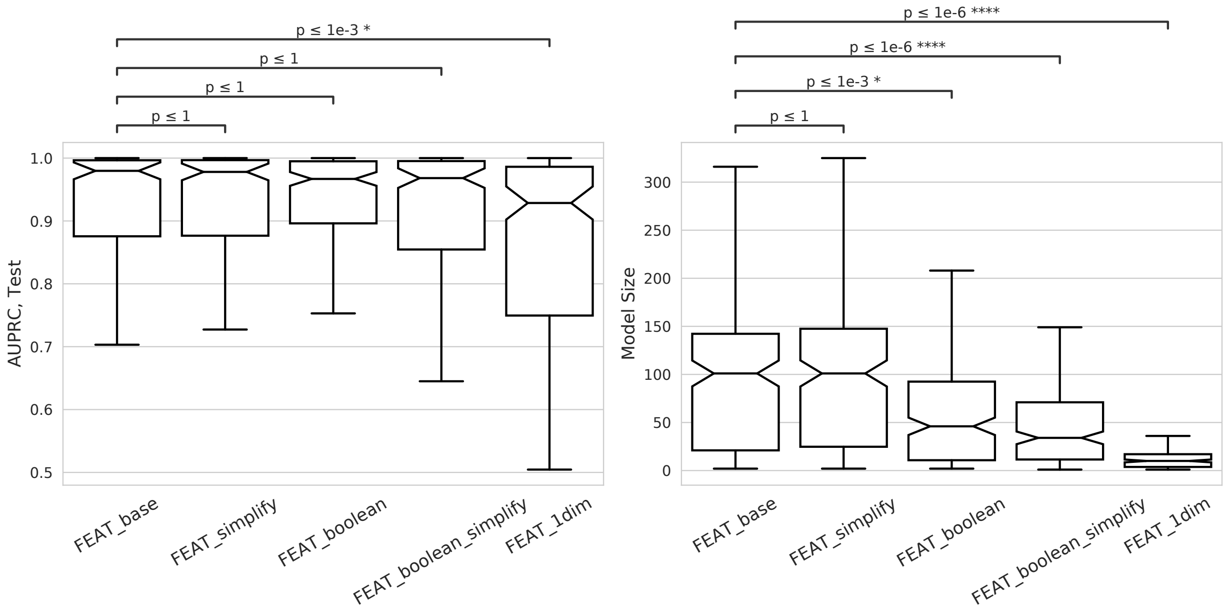

Supplementary Figure 1: Evaluation of FEAT modifications. *(Left)* Test AUPRC and *(Right)* model sizes of FEAT variants on 20 PMLB benchmark classification problems. Boxplots represent distribution of the 5-fold cross-validation mean test scores over 50 repeat realizations of the experiment. *p* values are calculated according to a Wilcoxon rank-sum test. Box centerline: median; box limits: quartiles; whiskers: 1.5x the interquartile range.

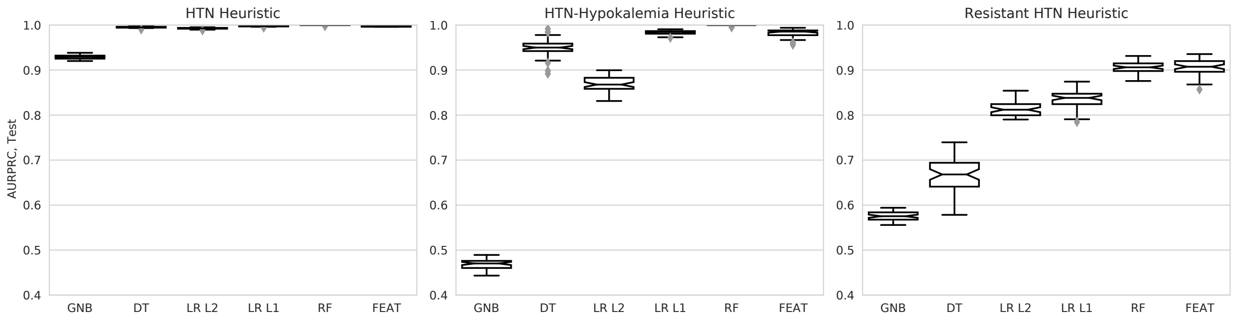

Supplementary Figure 2: Estimating model discrimination by cross-validation for expert heuristics. AUPRC scores for models trained in 5-fold cross-validation over 50 iterations, each averaged across testing folds. Each subplot represents a different training instance for an expert-curated heuristic. Box centerline: median; box limits: quartiles; whiskers: 1.5x the interquartile range; diamonds: outliers.

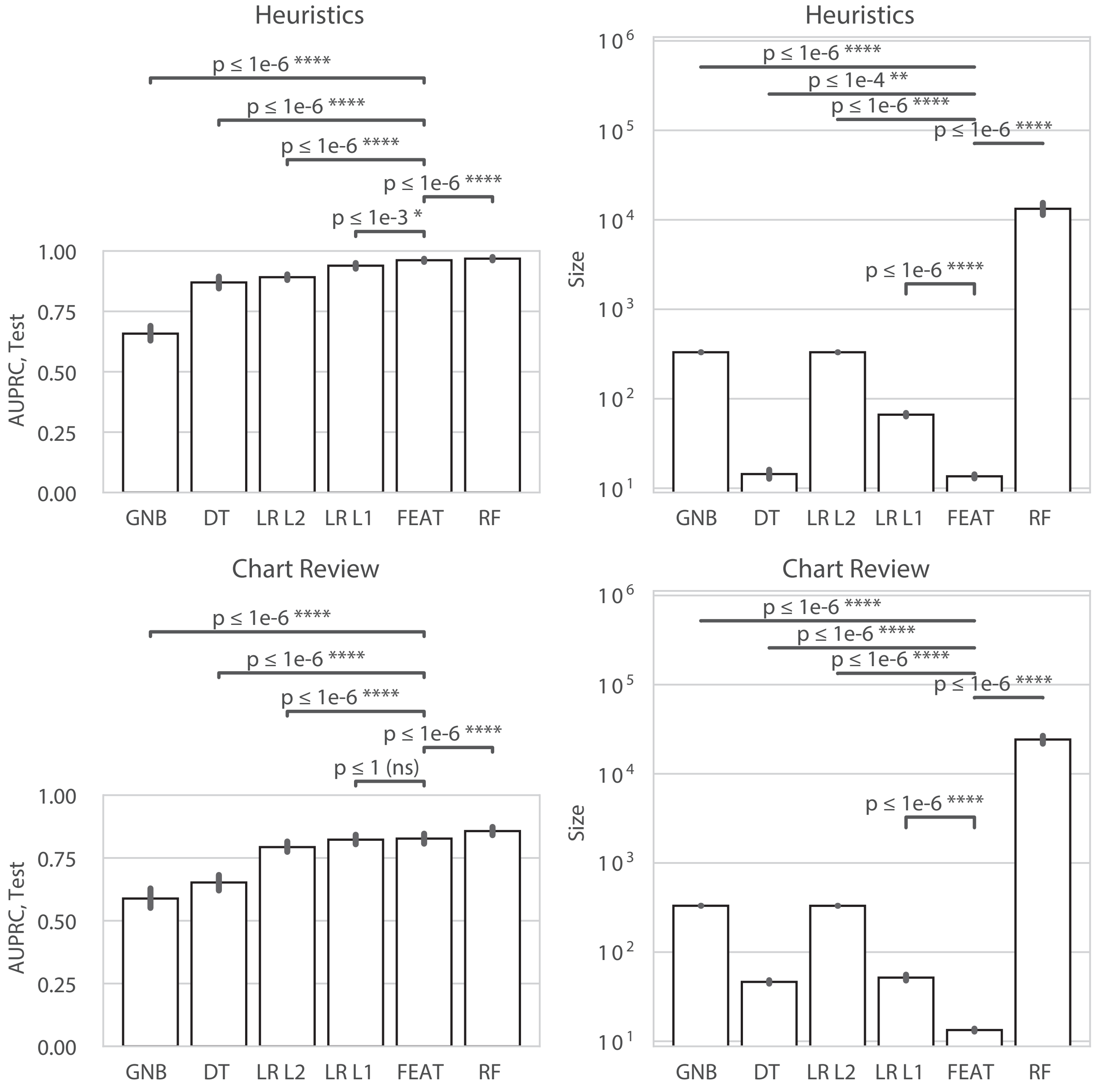

Supplementary Figure 3: Comparison of discrimination and size of models trained to learn heuristics and chart-reviewed phenotypes. Top plots indicate the median values of methods according to AUPRC (left) and model size (right), when tasked with predicting the three expert heuristics. On the bottom, equivalent plots are shown for predicting the chart-review phenotypes. Performance is ranked according to mean 5-fold CV performance and error bars indicate the standard

error over 50 realizations of the experiment. p values are calculated according to pairwise Wilcoxon rank-sum tests, with $\alpha = 0.001$. Error bars denote 95% bootstrap confidence intervals.

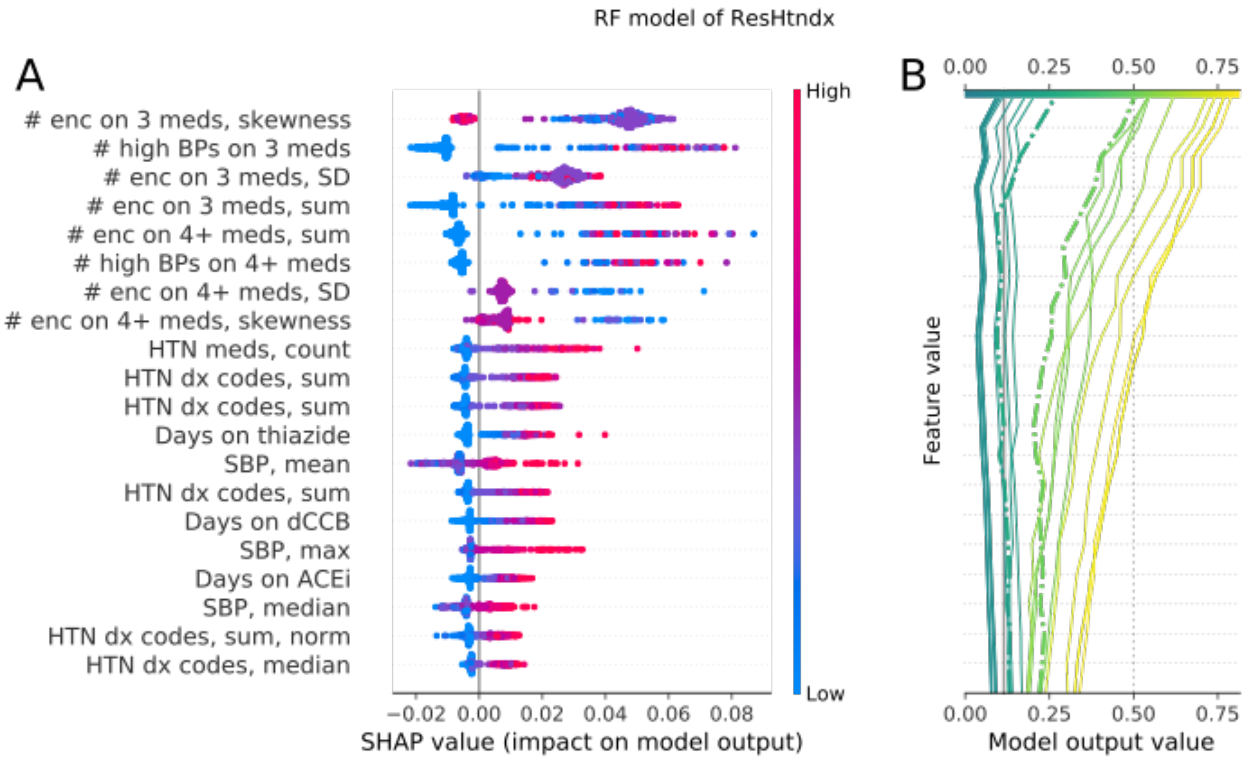

Supplementary Figure 4: **SHAP plots for random forest model trained to predict aTRH.** SHAP summary plots (A) and decision plots (B). The left plot indicates the most important features, ranked by the mean absolute SHAP value calculated on test data. The decision plot shows a sample of 10 positive and 10 negative point predictions by the models, with dotted lines indicating misclassifications. Note, the x-axis for the decision plot (B) is restricted to >= 0.

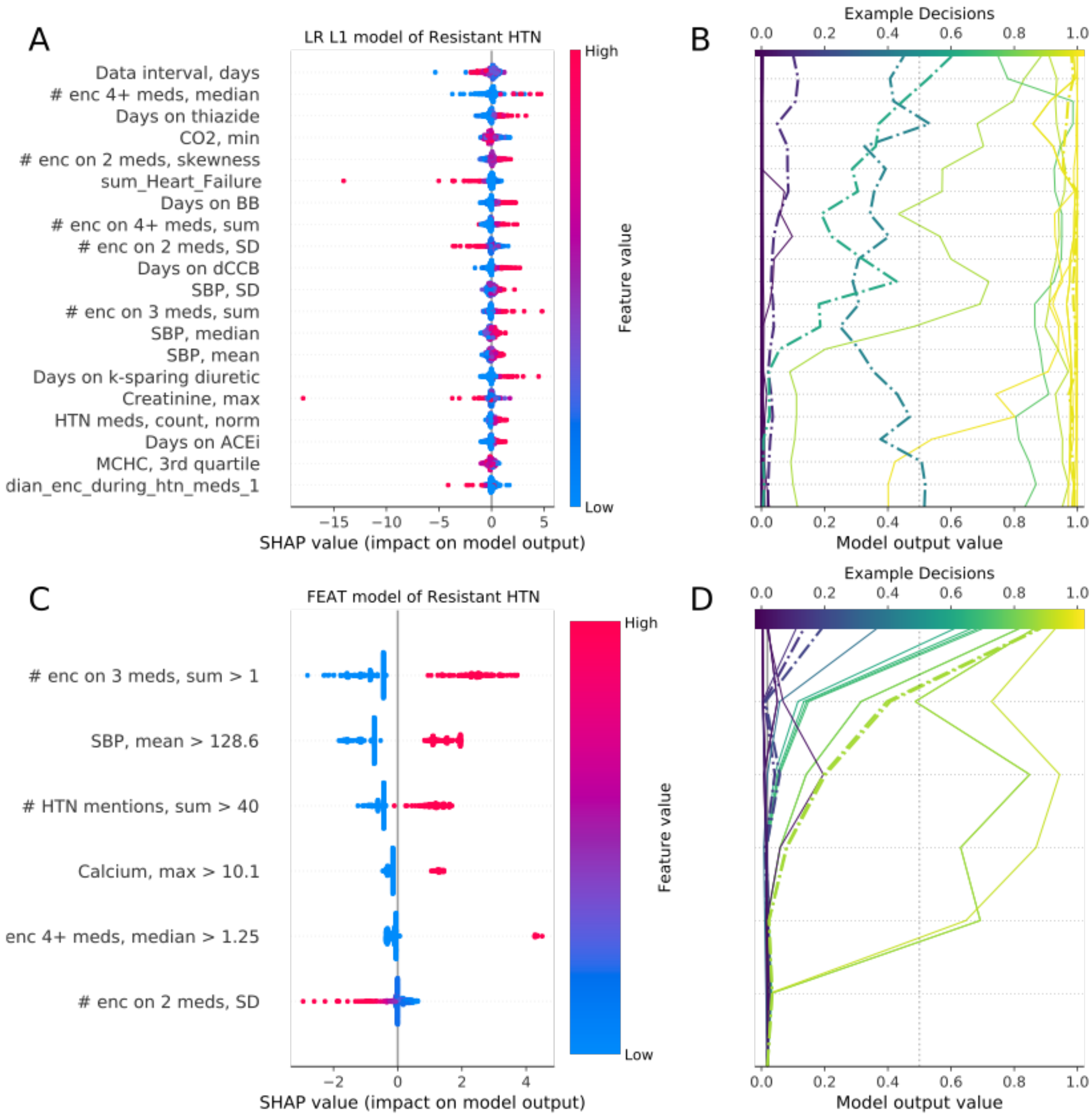

Supplementary Figure 5: **SHAP plots accounting for feature correlation for LR L1 and FEAT models trained to predict aTRH.** SHAP summary plots (A, C) and decision plots (B,D) according to the learned features of the LR L1 (top) and FEAT (bottom) models. In this case, SHAP values do not explicitly represent linear model coefficients. Instead, SHAP values are transformed by applying a linear projection to the input data and model coefficients, leading to feature importance estimates that

are more faithful to the data than the model. The left plots indicate the most important features, ranked by the mean absolute SHAP value calculated on test data. The decision plots show a sample of 10 positive and 10 negative point predictions by the models, with dotted lines indicating misclassifications. Note, the x-axis for the decision plots (B,D) are restricted to 0 – 1.

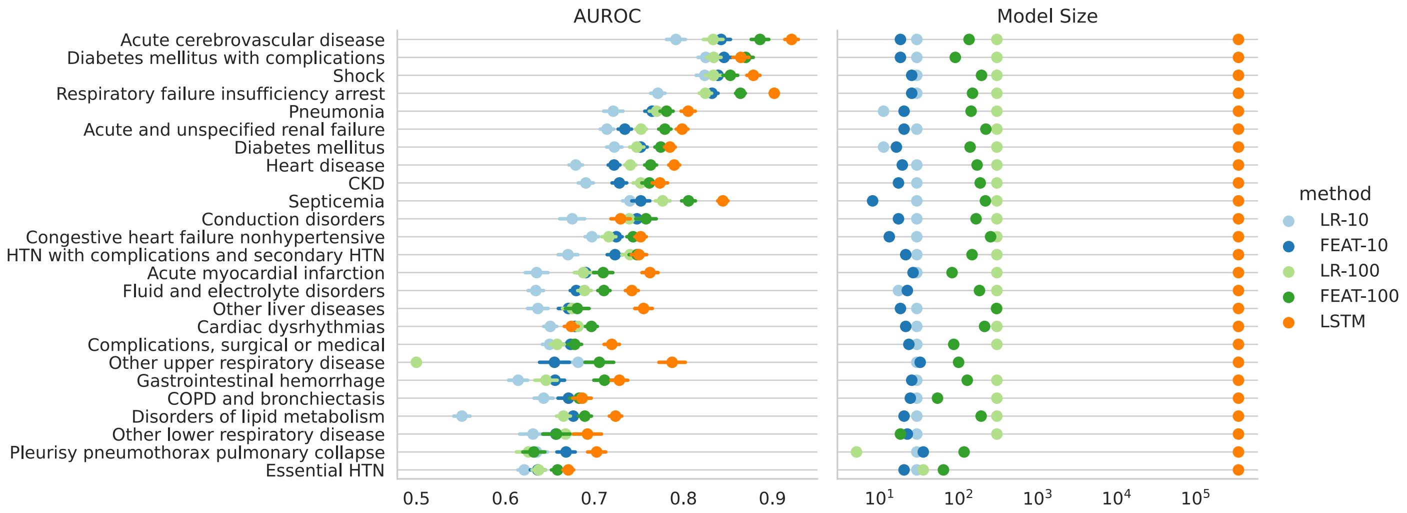

Supplementary Figure 6: **Phenotype-specific comparisons of methods on MIMIC-III benchmarks.** Performances for each phenotyping task and method are shown. Points indicate median value with bootstrap-estimated 95% confidence intervals shown as error bars. The left graph shows model AUROC, whereas the right shows model size. Note that for LSTMs, model size is calculated as the number of network parameters, and is therefore lower than the actual model size.

Supplementary Tables

Supplementary Table 1: FEAT method variants tested in benchmark experiment.

Common settings	population size = 500, generations = 200, max_time = 1 hour
Feat_base	FEAT with default arguments
Feat_simplify*	FEAT with post-run simplification
Feat_boolean	FEAT restricted to Boolean operators
Feat_boolean_simplify	FEAT restricted to Boolean operators with post-run simplification
Feat_1dim	FEAT restricted to producing a single feature (one dimensional)

Supplementary Table 2: Datasets from PMLB[16] used for benchmark comparisons.

**Dataset Name**	**Number of Features**	**Number of Instances**
Hill_Valley_with_noise	100	1212
Hill_Valley_without_noise	100	1212
backache	32	180
breast-cancer-wisconsin	30	569
chess	36	3196
clean1	168	476
clean2	168	6598
coil2000	85	9822
colic	22	368
dis	29	3772
horse-colic	22	368
hypothyroid	25	3163
ionosphere	34	351
kr-vs-kp	36	3196
molecular-biology_promoters	58	106
sonar	60	208
spambase	57	4601
spectf	44	349
tokyo1	44	959
wdbc	30	569

Supplementary Table 3: Cross-validation results for models trained to predict the expert-curated heuristics.

Phenotype	Method	Median CV AUPRC (IQR)	Median CV AUROC (IQR)	Median Size (IQR)
HTN Heuristic	GNB	0.93 (+-0.01)	0.96 (+-0.00)	331.00 (+-0.00)
	DT	0.99 (+-0.00)	1.00 (+-0.00)	**4.60 (+-0.00)**
	LR L1	1.00 (+-0.00)	1.00 (+-0.00)	52.30 (+-5.90)
	LR L2	0.99 (+-0.00)	0.99 (+-0.00)	330.80 (+-0.00)
	RF	**1.00 (+-0.00)**	**1.00 (+-0.00)**	8760.40 (+-6278.30)
	FEAT	1.00 (+-0.00)	1.00 (+-0.00)	8.20 (+-0.95)
HTN-Hypokalemia Heuristic	GNB	0.47 (+-0.02)	0.86 (+-0.00)	331.00 (+-0.00)
	DT	0.95 (+-0.02)	0.99 (+-0.01)	**10.80 (+-1.60)**
	LR L1	0.98 (+-0.01)	1.00 (+-0.00)	65.10 (+-18.05)
	LR L2	0.87 (+-0.02)	0.96 (+-0.01)	330.80 (+-0.00)
	RF	**1.00 (+-0.00)**	**1.00 (+-0.00)**	1855.80 (+-1640.80)
	FEAT	0.99 (+-0.01)	1.00 (+-0.00)	18.30 (+-2.55)
Resistant HTN Heuristic	GNB	0.58 (+-0.02)	0.92 (+-0.01)	331.00 (+-0.00)
	DT	0.67 (+-0.05)	0.89 (+-0.03)	27.00 (+-3.20)
	LR L1	0.84 (+-0.02)	0.95 (+-0.01)	70.50 (+-16.90)
	LR L2	0.81 (+-0.02)	0.94 (+-0.01)	330.80 (+-0.00)
	RF	**0.91 (+-0.02)**	**0.99 (+-0.00)**	27235.00 (+-13392.00)
	FEAT	**0.91 (+-0.02)**	0.98 (+-0.01)	**14.20 (+-1.70)**

Best performances and smallest size (p<0.001) are shown in bold.

Supplementary Table 4. Model discrimination and size for each target phenotype in training cross-validation.

Phenotype	Method	Median CV AUPRC (IQR)	Median CV AUROC (IQR)	Median Size (IQR)
HTN Diagnosis	GNB	0.93 (+-0.00)	0.95 (+-0.00)	331.00 (+-0.00)
	DT	0.91 (+-0.01)	0.94 (+-0.01)	51.00 (+-5.20)
	LR L1	0.98 (+-0.00)	0.98 (+-0.00)	23.20 (+-5.50)
	LR L2	0.97 (+-0.00)	0.98 (+-0.00)	330.80 (+-0.00)
	RF	**0.98 (+-0.00)**	**0.99 (+-0.00)**	20397.60 (+-19474.10)
	FEAT	0.98 (+-0.00)	0.98 (+-0.00)	**13.90 (+-2.90)**
HTN-Hypokalemia Diagnosis	GNB	0.38 (+-0.01)	0.86 (+-0.02)	331.00 (+-0.00)
	DT	0.63 (+-0.06)	0.88 (+-0.04)	30.20 (+-2.40)
	LR L1	0.80 (+-0.02)	0.94 (+-0.02)	68.10 (+-15.30)
	LR L2	0.73 (+-0.02)	0.91 (+-0.02)	330.80 (+-0.00)
	RF	**0.84 (+-0.02)**	**0.98 (+-0.01)**	13443.40 (+-8241.30)
	FEAT	0.82 (+-0.03)	0.96 (+-0.01)	**16.20 (+-2.70)**
Resistant HTN Diagnosis	GNB	0.46 (+-0.02)	0.90 (+-0.02)	331.00 (+-0.00)
	DT	0.41 (+-0.06)	0.78 (+-0.04)	56.80 (+-7.40)
	LR L1	0.69 (+-0.04)	0.93 (+-0.02)	52.90 (+-17.95)
	LR L2	0.69 (+-0.04)	0.91 (+-0.01)	330.80 (+-0.00)
	RF	**0.75 (+-0.02)**	**0.96 (+-0.00)**	38835.00 (+-20521.10)
	FEAT	0.69 (+-0.05)	0.94 (+-0.01)	**9.80 (+-1.80)**

Best performances and smallest size (p<0.001) are shown in bold. Corresponds to the results of Figure 2 in the main text.

Supplementary Table 5: Internal-external model performance.

PHENOTYPE	METHOD	TEST BAL. LOG LOSS	TEST AUPRC	TEST AUROC	SIZE
**HTN HEURISTIC**	GNB	2.73 (1.77-3.83)	0.93 (0.88-0.97)	0.96 (0.93-0.98)	331
	DT	0.13 (0.0-0.39)	0.99 (0.98-1.0)	1.0 (0.99-1.0)	3
	LR L1	0.08 (0.05-0.1)	1.0 (1.0-1.0)	1.0 (1.0-1.0)	16
	LR L2	0.08 (0.05-0.11)	1.0 (1.0-1.0)	1.0 (1.0-1.0)	331
	RF	0.03 (0.02-0.04)	1.0 (1.0-1.0)	1.0 (1.0-1.0)	25166
	FEAT	0.04 (0.0-0.12)	0.99 (0.98-1.0)	1.0 (0.99-1.0)	2
**HTN-HYPOKALEMIA HEURISTIC**	GNB	7.88 (5.61-10.05)	0.5 (0.39-0.61)	0.83 (0.78-0.88)	331
	DT	1.5 (0.31-2.92)	0.93 (0.87-0.98)	0.96 (0.92-0.99)	21
	LR L1	0.04 (0.02-0.09)	0.99 (0.97-1.0)	1.0 (0.99-1.0)	80
	LR L2	0.27 (0.15-0.41)	0.94 (0.89-0.98)	0.99 (0.97-0.99)	331
	RF	0.03 (0.02-0.04)	1.0 (1.0-1.0)	1.0 (1.0-1.0)	1364
	FEAT	0.07 (0.03-0.11)	0.98 (0.95-1.0)	1.0 (0.99-1.0)	22
**RESISTANT HTN HEURISTIC**	GNB	4.29 (2.78-6.03)	0.66 (0.55-0.78)	0.92 (0.89-0.95)	331
	DT	4.07 (2.3-6.09)	0.73 (0.62-0.83)	0.88 (0.83-0.93)	37
	LR L1	0.75 (0.33-1.25)	0.9 (0.83-0.96)	0.96 (0.92-0.99)	140
	LR L2	0.38 (0.2-0.62)	0.91 (0.84-0.97)	0.96 (0.92-0.99)	331
	RF	0.14 (0.12-0.17)	0.95 (0.9-0.99)	0.99 (0.98-1.0)	2984
	FEAT	0.13 (0.08-0.2)	0.96 (0.92-0.99)	0.99 (0.98-1.0)	17
**HTN DIAGNOSIS**	GNB	3.15 (2.08-4.26)	0.92 (0.87-0.96)	0.94 (0.91-0.97)	331
	DT	1.12 (0.48-1.89)	0.95 (0.91-0.98)	0.96 (0.94-0.98)	67
	LR L1	0.17 (0.12-0.24)	0.98 (0.95-1.0)	0.98 (0.97-0.99)	30
	LR L2	0.22 (0.14-0.31)	0.98 (0.96-0.99)	0.98 (0.96-0.99)	331
	RF	0.14 (0.1-0.19)	0.99 (0.98-1.0)	0.99 (0.98-1.0)	122402
	FEAT	0.14 (0.09-0.21)	0.99 (0.98-1.0)	0.99 (0.98-1.0)	15
**HTN-HYPOKALEMIA DIAGNOSIS**	GNB	7.47 (5.2-9.82)	0.45 (0.33-0.57)	0.85 (0.8-0.9)	331
	DT	4.57 (2.47-7.01)	0.63 (0.48-0.76)	0.86 (0.8-0.92)	49
	LR L1	0.32 (0.21-0.43)	**0.91 (0.83-0.96)**	0.98 (0.96-0.99)	31
	LR L2	0.44 (0.23-0.68)	**0.85 (0.76-0.93)**	0.95 (0.91-0.98)	331
	RF	0.36 (0.29-0.43)	**0.88 (0.8-0.95)**	0.97 (0.95-0.99)	7568
	FEAT	0.26 (0.18-0.36)	**0.92 (0.85-0.96)**	0.98 (0.97-0.99)	17
**RESISTANT HTN DIAGNOSIS**	GNB	5.45 (3.56-7.29)	0.55 (0.43-0.66)	0.89 (0.86-0.93)	331
	DT	6.14 (3.86-8.44)	0.51 (0.36-0.64)	0.79 (0.72-0.86)	77
	LR L1	0.42 (0.32-0.53)	**0.78 (0.65-0.89)**	0.95 (0.92-0.97)	50
	LR L2	0.43 (0.3-0.58)	**0.74 (0.6-0.86)**	0.93 (0.88-0.96)	331
	RF	0.37 (0.31-0.45)	**0.85 (0.76-0.93)**	0.97 (0.94-0.98)	161682
	FEAT	0.38 (0.29-0.49)	**0.77 (0.63-0.89)**	0.95 (0.92-0.97)	12

Discrimination and size for each target phenotype in simulation where training and testing data are split based on primary care practice site.

Supplementary Table 6: Method comparisons on MIMIC-III phenotyping tasks.

	Macro AUPRC	Macro AUROC	Model Size
**LR-10**	**0.30 (0.09-0.52)**	**0.68 (0.56-0.83)**	**27.1 (11-29)**
**FEAT-10**	**0.35 (0.11-0.55)**	**0.72 (0.64-0.86)**	**21 (8-35)**
**LR-100**	**0.37 (0.12-0.57)**	**0.72 (0.58-0.84)**	**265 (1-299)**
**FEAT-100**	**0.38 (0.13-0.61)**	**0.74 (0.65-0.88)**	**148 (18-296)**
**LSTM**	**0.41 (0.16-0.67)**	**0.77 (0.67-0.91)**	341,249 parameters

Macro-averaged AUROC, AUPRC, and model size comparisons across 25 phenotyping tasks in MIMIC-II are shown. Bootstrapped 95% confidence intervals (CIs) shown in parenthesis. Bold indicates best and those with an overlapping CI.

Supplementary Table 7: EHR features considered as potential predictors

Group	VARIABLE	VARIABLE DESCRIPTION
Identifiers	UNI_ID	the unique study-generated identifier for Patient
Demo	age	patient's age at right-censoring date
	Male	patient's indicated Sex (1 = male, 0 = female)
	BLACK	1 = black, 0 = non-black
	OTHER	1 = asian, other, mixed,native american,pacific islander, 0 = black or white
	WHITE	1 = white, 0 = non-white
	ZIP_CAT	distance from patient's home to 19104, in category
Encounter	MASTER_LOCATION_CODE	code for healthcare site (not one hot encoded), common service for all source systems. This is used to map UPHS's various versions of the same or similar codes into a matched list of services. This data will persist to the MDM level.
	GENERAL_INTERNAL_MEDICINE	1 = IM practice, 0 = FM practice
BMI/Weight	weight_min/max/median/sd/skewness	min/max/median/sd/skewness of weights
	bmi_min/max/sd/skewness	min/max/sd/skewness of BMI
BP	bp_n	total number of bp measurements
	min_systolic	minimum of systolic blood pressure measured
	min_diastolic	minimum of diastolic blood pressure measured
	max_systolic	maximum of systolic blood pressure measured
	max_systolic	maximum of diastolic blood pressure measured
	mean_systolic	mean of systolic blood pressure measured
	mean_diastolic	mean of diastolic blood pressure measured
	median_systolic	median of systolic blood pressure measured
	median_diastolic	median of diastolic blood pressure measured
	sd_systolic	standard deviation of systolic blood pressure measured
	sd_diastolic	standard deviation of diastolic blood pressure measured
	skew_systolic	skewness of systolic blood pressure measured
	skew_diastolic	skewness of diastolic blood pressure measured
	high_bp_n	number of high blood pressure, SBP >= 140 or DBP >= 90
	mean_high_bp_systolic	mean systolic bp of all high blood pressure measurements (SBP >=140 or DBP >=90)
	mean_high_bp_diastolic	mean diastolic bp of all high blood pressure measurements (SBP >=140 or DBP >=90)

	median_high_bp_systolic	median systolic bp of all high blood pressure measurements (SBP >=140 or DBP >=90)
	median_high_bp_diastolic	median diastolic bp of all high blood pressure measurements (SBP >=140 or DBP >=90)
	sd_high_bp_systolic	standard deviation of systolic bp of all high blood pressure measurements (SBP >=140 or DBP >=90)
	sd_high_bp_diastolic	standard deviation of diastolic bp of all high blood pressure measurements (SBP >=140 or DBP >=90)
	skew_high_bp_systolic	skewness of systolic bp of all high blood pressure measurements (SBP >=140 or DBP >=90)
	skew_high_bp_diastolic	skewness of diastolic bp of all high blood pressure measurements (SBP >=140 or DBP >=90)
	median/sd/skew_high_bp_n_yr	median/sd/skewness of high blood pressure measurements (SBP >=140 or DBP >=90) per year
**Labs**	max.lab_XXX	maximum of XXX lab test
	min.lab_XXX	minimum of XXX lab test
	median.lab_XXX	median of XXX lab test
	q1.lab_XXX	1st quantile of XXX lab test
	q3.lab_XXX	3rd quantile of XXX lab test
**Dx**	median_ICD_XXX (Dx)	median XXX ICD-9 and ICD-10 codes, by year
	sum_ICD_XXX (Dx)	sum XXX ICD-9 and ICD-10 codes, by year
	median_XXX (disease name)	median XXX disease name, by year
	sum_XXX (disease name)	sum XXX disease name, year
	Dx_N	number of total ICD-9 and ICD-10 codes (PK_DX_ID)
	enc_N	number of OUTPATIENT (including INFUSION VISIT) encounters
	dx_days_x	days from 1st Dx to last Dx in system
**Medication**	HTN_MED_days_XXX	days on med XXX (including anti-HTN and Potassium Supplement)
	MED_N	number of medication prescriptions total
	high_BP_during_htn_meds_1/2/3/4_plus	number of high BP measurements during 1/2/3/4+ anti-HTN meds
	sum_enc_during_htn_meds_1/2/3/4_plus	number of OUTPATIENT encounters during 1/2/3/4+ meds
	median_enc_during_htn_meds_1/2/3/4_plus	median number (by year) of OUTPATIENT encounters during 1/2/3/4+ meds
	sd_enc_during_htn_meds_1/2/3/4_plus	sd of number (by year) of OUTPATIENT encounters during 1/2/3/4+ meds
	skewness_enc_during_htn_meds_1/2/3/4_plus	skewness of number (by year) of OUTPATIENT encounters during 1/2/3/4+ meds
	N_med_K_chlo_enc	number of encounters on POTASSIUM_CHLORIDE/POTASSIUM_GLUCONATE
	sd_med_K_chlo_enc	sd of number (by year) of encounters on POTASSIUM_CHLORIDE/POTASSIUM_GLUCONATE
	skewness_med_K_chlo_enc	skewness of number (by year) of encounters on POTASSIUM_CHLORIDE/POTASSIUM_GLUCONATE
**Heuristic Features**	low_K_N	# of low potassium test results
	test_K_N	# of potassium test results
	Med_Potassium_N	# of potassium supplement medication subscriptions
	Dx_HypoK_N	# of Hypokalemia Dx
**HTN Score Features**	ICD_hyp_sum	HTN ICD codes
	MED_HTN_N	anti-HTN med prescriptions
	bp_hyp_norm	high_bp_n/bp_n
	ICD_hyp_sum_norm	ICD_hyp_sum/Dx_N
	MED_HTN_N_norm	MED_HTN_N/MED_N
	re_hyp_spe_norm	re_htn_spec/words_n

**Regex**	re_htn_sum	sum of regex counts in clinical notes for hypertension
	re_htn_spec_sum	sum of regex counts in clinical notes for hypertension (specific, excluding preliminary negations)
	re_htn_teixera_sum	sum of regex counts in clinical notes for hypertension (regex used in Teixeira paper)
	re_word_count_sum	sum word counts in clinical notes
	re_htn_max	maximum of regex counts in clinical notes for hypertension
	re_htn_spec_max	maximum of regex counts in clinical notes for hypertension (specific, excluding preliminary negations)
	re_htn_teixera_max	maximum of regex counts in clinical notes for hypertension (regex used in Teixeira paper)
	re_word_count_max	maximum word counts in clinical notes
	re_htn_mean	mean of regex counts in clinical notes for hypertension
	re_htn_spec_mean	mean of regex counts in clinical notes for hypertension (specific, excluding preliminary negations)
	re_htn_teixera_mean	mean of regex counts in clinical notes for hypertension (regex used in Teixeira paper)
	re_word_count_mean	mean word counts in clinical notes
	re_htn_median	median of regex counts in clinical notes for hypertension
	re_htn_spec_median	median of regex counts in clinical notes for hypertension (specific, excluding preliminary negations)
	re_htn_teixera_median	median of regex counts in clinical notes for hypertension (regex used in Teixeira paper)
	re_word_count_median	median word counts in clinical notes
	re_htn_sd	standard deviation of regex counts in clinical notes for hypertension
	re_htn_spec_sd	standard deviation of regex counts in clinical notes for hypertension (specific, excluding preliminary negations)
	re_htn_teixera_sd	standard deviation of regex counts in clinical notes for hypertension (regex used in Teixeira paper)
	re_word_count_sd	standard deviation of word counts in clinical notes
	re_htn_skewness	skewness of regex counts in clinical notes for hypertension
	re_htn_spec_skewness	skewness of regex counts in clinical notes for hypertension (specific, excluding preliminary negations)
	re_htn_teixera_skewness	skewness of regex counts in clinical notes for hypertension (regex used in Teixeira paper)
	re_word_count_skewness	skewness of word counts in clinical notes

Supplementary Table 8: EHR laboratory results considered as predictors

Labs
Pct.BASOPHILS
Pct.EOSINOPHILS
Pct.LYMPHOCYTES
Pct.MONOCYTES
Pct.NEUTROPHILS
ALBUMIN
ALKALINE.PHOSPHATASE
ALT
AST
BILIRUBIN.TOTAL
CALCIUM
CARBON.DIOXIDE
CHLORIDE
CHOLESTEROL
CHOLESTEROL.CALCULATED.LOW.DENSITY.LIPOPROTEIN
CHOLESTEROL.CALCULATED.HIGH.DENSITY.LIPOPROTEIN
CREATININE
HEMATOCRIT
HEMOGLOBIN
MEAN.CELLULAR.HEMOGLOBIN
MEAN.CELLULAR.HEMOGLOBIN.CONCENTRATION
MEAN.CELLULAR.VOLUME
PLATELETS
POTASSIUM
PROTEIN.TOTAL
RDW
RED.BLOOD.CELLS
SODIUM
THYROID.STIMULATING.HORMONE
TRIGLYCERIDES
UREA.NITROGEN
WBC

Supplementary Table 9: EHR diagnosis codes considered as predictors, encoded as median count per year

**median_ICD_XXX (Dx)**
median_E03_9
median_E11_9
median_E78_00
median_E78_01
median_E78_2
median_E78_5
median_I10
median_I16_0
median_I16_1
median_I16_9
**median_XXX (disease name)**
median_Diabetes_type_1
median_Dyslipidemias
median_Essential_HTN
median_HTN_Emergency
median_Hypothyroidism

Supplementary Table 10: EHR diagnosis codes considered as predictors, encoded as total count

**sum_ICD_XXX (Dx)**
sum_E03_8
sum_E03_9
sum_E11_65
sum_E11_9
sum_E66_01
sum_E66_09
sum_E66_1
sum_E66_8
sum_E66_9
sum_E78_00
sum_E78_01
sum_E78_2
sum_E78_5
sum_E87_6
sum_G47_30
sum_G47_33
sum_I10
sum_I16_0
sum_I16_1
sum_I16_9
sum_I25_10
sum_I48_0
sum_I48_1
sum_I48_2
sum_I48_91
sum_L70_8
sum_N18_3
**sum_XXX (disease name)**
sum_ACNE
sum_Arrythmias
sum_Atrial_fibrillation
sum_CAD_native
sum_CKD
sum_Diabetes_type_2
sum_Dyslipidemias

sum_Essential_HTN
sum_Heart_Failure
sum_HTN_Emergency
sum_Hypokalemia
sum_Hypothyroidism
sum_Obesity
sum_Obstructive_Sleep_Apnea

Supplementary Table 11: Anti-hypertensive medication features considered, encoded as number of days prescribed

**HTN_MED_days_XXX**
HTN_MED_days_ACEI_ARB
HTN_MED_days_ALDOSTERONE_ANTAGONIST
HTN_MED_days_ALDOSTERONE_ANTAGONISTS
HTN_MED_days_ALPHA_ANTAGONISTS
HTN_MED_days_BETA_BLOCKERS
HTN_MED_days_CENTRAL_ALPHA_AGONISTS
HTN_MED_days_DIHYDRO_CCBS
HTN_MED_days_HYDRALAZINE
HTN_MED_days_K_SPARING_DIURETICS
HTN_MED_days_LOOP_DIURETICS
HTN_MED_days_MINOXIDIL
HTN_MED_days_NON_DIHYDRO_CCBS
HTN_MED_days_RENIN_ANTAGONIST
HTN_MED_days_THIAZIDE
HTN_MED_days_POTASSIUM_CHLORIDE

Supplementary Table 12: EHR features included in trained computable phenotypes

Full Name	Short Name
**Days between 1st dx to last dx code**	Data interval, days
**Number of high BP measurements while on 3 anti-HTN meds**	# high BPs on 3 meds
**Number of high BP measurements while on 4+ anti-HTN meds**	# high BPs on 4+ meds
**Days prescribed ACE inhibitors**	Days on ACEi
**Days prescribed beta blockers**	Days on BB
**Days prescribed dihydropyridine calcium channel blockers**	Days on dCCB
**Days prescribed potassium sparing diuretics**	Days on k-sparing diuretic
**Days prescribed thiazides**	Days on thiazide
**Sum of HTN ICD codes**	HTN dx codes, sum
**Sum of HTN ICD codes divided by the total number of ICD codes**	HTN dx codes, sum, norm
**Maximum of systolic blood pressure measured**	SBP, max
**Maximum of calcium measured**	Calcium, max
**Maximum of creatinine measured**	Creatinine, max
**Mean of systolic blood pressure measured**	SBP, mean
**Number of anti-hypertension medication prescriptions**	HTN meds, count
**Number of anti-hypertension medication prescriptions divided by the total number of prescribed medications**	HTN meds, count, norm
**Median number (by year) of OUTPATIENT encounters during 4+ anti-hypertension medications**	# enc 4+ meds, median
**Count of I10 (hypertension) ICD codes, median per year**	HTN dx codes, median
**Median of systolic blood pressure measured**	SBP, median
**Median of potassium measured**	K, median
**Minimum of potassium measured**	K, min
**Sum of regex counts in clinical notes for hypertension**	# HTN mentions, sum
**Standard deviation of number (by year) of OUTPATIENT encounters during 2 anti-hypertension medications**	# enc on 2 meds, SD
**Standard deviation of number (by year) of OUTPATIENT encounters during 3 anti-hypertension medications**	# enc on 3 meds, SD
**Standard deviation of number (by year) of OUTPATIENT encounters during 4+ anti-hypertension medications**	# enc on 4+ meds, SD
**Standard deviation of systolic blood pressure measured**	SBP, SD
**Skewness of number (by year) of OUTPATIENT encounters during 2 anti-hypertension medications**	# enc on 2 meds, skewness

**Skewness of number (by year) of OUTPATIENT encounters during 3 anti-hypertension medications**	# enc on 3 meds, skewness
**Skewness of number (by year) of OUTPATIENT encounters during 4+ anti-hypertension medications**	# enc on 4+ meds, skewness
**Sum of number (by year) of OUTPATIENT encounters during 3 anti-hypertension medications**	# enc on 3 meds, sum
**Sum of number (by year) of OUTPATIENT encounters during 4+ anti-hypertension medications**	# enc on 4+ meds, sum
**Sum of I10 (hypertension) ICD codes**	HTN dx codes, sum
**Sum of I10 (hypertension) ICD codes**	HTN dx codes, sum
**High Density Lipoprotein (HDL) cholesterol, min**	HDLc, min
**High Density Lipoprotein (HDL) cholesterol, max**	HDLc, max
**Chloride, 1st quartile**	Chloride, min
**Carbon dioxide, min**	CO2, min
**Mean cellular hemoglobin concentration (MCHC), 3rd quartile**	MCHC, 3rd quartile
**Platelets, max**	Platelets, max
**Mean cellular hemoglobin concentration (MCHC), 1st quartile**	MCHC, 1st quartile
**Low Density Lipoprotein (LDL) cholesterol, calculated, median**	LDLc, median
**Regex counts in clinical notes for hypertension, median per year**	# of HTN mentions, median
**Albumin, min**	Albumin, min
**Calcium, 1st quartile**	Calcium, 1st quartile
**Chloride, median**	Chloride, median
**Thyroid stimulating hormone (TSH), 1st quartile**	TSH, 1st quartile

References

1. Guidotti R, Monreale A, Ruggieri S, Turini F, Giannotti F, Pedreschi D. A Survey of Methods for Explaining Black Box Models. *ACM Computing Surveys*. 2018;51(5):93:1-93:42. doi:10.1145/3236009

2. Breiman L. Random forests. *Machine learning*. 2001;45(1):5-32.

3. La Cava W, Bauer CR, Moore JH, Pendergrass SA. Interpretation of machine learning predictions for patient outcomes in electronic health records. In: AMIA 2019 Annual Symposium. AMIA; 2019. https://arxiv.org/abs/1903.12074

4. Ribeiro MT, Singh S, Guestrin C. Why should i trust you?: Explaining the predictions of any classifier. In: *Proceedings of the 22nd ACM SIGKDD International Conference on Knowledge Discovery and Data Mining*. ACM; 2016:1135-1144.

5. Lundberg SM, Lee SI. A Unified Approach to Interpreting Model Predictions. In: Guyon I, Luxburg UV, Bengio S, et al., eds. *Advances in Neural Information Processing Systems 30*. Curran Associates, Inc.; 2017:4765-4774. Accessed November 22, 2019. http://papers.nips.cc/paper/7062-a-unified-approach-to-interpreting-model-predictions.pdf

6. Lundberg SM, Nair B, Vavilala MS, et al. Explainable machine-learning predictions for the prevention of hypoxaemia during surgery. *Nat Biomed Eng*. 2018;2(10):749-760. doi:10.1038/s41551-018-0304-0

7. Murdoch WJ, Singh C, Kumbier K, Abbasi-Asl R, Yu B. Definitions, methods, and applications in interpretable machine learning. *Proceedings of the National Academy of Sciences*. 2019;116(44):22071-22080. doi:10.1073/pnas.1900654116

8. Tibshirani R. Regression shrinkage and selection via the lasso. *Journal of the Royal Statistical Society Series B (Methodological)*. Published online 1996:267-288.

9. Hoerl AE, Kennard RW. Ridge regression: Biased estimation for nonorthogonal problems. *Technometrics*. 1970;12(1):55-67.

10. Ching T, Himmelstein DS, Beaulieu-Jones BK, et al. Opportunities And Obstacles For Deep Learning In Biology And Medicine. *bioRxiv*. Published online May 28, 2017:142760. doi:10.1101/142760

11. Smits GF, Kotanchek M. Pareto-Front Exploitation in Symbolic Regression. In: O’Reilly UM, Yu T, Riolo R, Worzel B, eds. *Genetic Programming Theory and Practice II*. Genetic Programming. Springer US; 2005:283-299. doi:10.1007/0-387-23254-0_17

12. Koza JR. *Genetic Programming: On the Programming of Computers by Means of Natural Selection*. MIT Press; 1992.

13. Schmidt M, Lipson H. Distilling free-form natural laws from experimental data. *Science*. 2009;324(5923):81-85.

14. Schmidt MD, Vallabhajosyula RR, Jenkins JW, et al. Automated refinement and inference of analytical models for metabolic networks. *Physical Biology*. 2011;8(5):055011. doi:10.1088/1478-3975/8/5/055011

15. La Cava W, Danai K, Spector L, Fleming P, Wright A, Lackner M. Automatic identification of wind turbine models using evolutionary multiobjective optimization. *Renewable Energy*. 2016;87, Part 2:892-902. doi:10.1016/j.renene.2015.09.068

16. Olson RS, La Cava W, Orzechowski P, Urbanowicz RJ, Moore JH. PMLB: A Large Benchmark Suite for Machine Learning Evaluation and Comparison. *BioData Mining*. Published online 2017. https://arxiv.org/abs/1703.00512
