# Supplementary material for: A flexible symbolic regression method for constructing interpretable clinical prediction models
Source: NPJ Digit Med. 2023 Jun 5;6:107. doi: 10.1038/s41746-023-00833-8 (PMC10241925; doi:10.1038/s41746-023-00833-8)
Supplement: Supplementary file 2 — Reporting Summary [file 41746_2023_833_MOESM2_ESM.pdf]

nature portfolio

Corresponding author(s): Daniel S. Herman

Last updated by author(s): 5/3/2023

Reporting Summary

Nature Portfolio wishes to improve the reproducibility of the work that we publish. This form provides structure for consistency and transparency in reporting. For further information on Nature Portfolio policies, see our Editorial Policies and the Editorial Policy Checklist.

Statistics

For all statistical analyses, confirm that the following items are present in the figure legend, table legend, main text, or Methods section.

n/a	Confirmed	
☐	☒	The exact sample size (*n*) for each experimental group/condition, given as a discrete number and unit of measurement
☐	☒	A statement on whether measurements were taken from distinct samples or whether the same sample was measured repeatedly
☐	☒	The statistical test(s) used AND whether they are one- or two-sided<br>*Only common tests should be described solely by name; describe more complex techniques in the Methods section.*
☐	☒	A description of all covariates tested
☐	☒	A description of any assumptions or corrections, such as tests of normality and adjustment for multiple comparisons
☐	☒	A full description of the statistical parameters including central tendency (e.g. means) or other basic estimates (e.g. regression coefficient) AND variation (e.g. standard deviation) or associated estimates of uncertainty (e.g. confidence intervals)
☐	☒	For null hypothesis testing, the test statistic (e.g. *F*, *t*, *r*) with confidence intervals, effect sizes, degrees of freedom and *P* value noted<br>*Give P values as exact values whenever suitable.*
☒	☐	For Bayesian analysis, information on the choice of priors and Markov chain Monte Carlo settings
☒	☐	For hierarchical and complex designs, identification of the appropriate level for tests and full reporting of outcomes
☐	☒	Estimates of effect sizes (e.g. Cohen's *d*, Pearson's *r*), indicating how they were calculated

Our web collection on statistics for biologists contains articles on many of the points above.

Software and code

Policy information about availability of computer code

Data collection	Electronic health record data was extracted from the Penn Medicine Penn Data Store and EPIC Clarity databases. Data was extracted and processed using code in the linked ehr_transform repository (https://bitbucket.org/hermanlab/ehr_transform/). Code for MIMIC-III analyses is in a dedicated repository (https://github.com/cavalab/mimic3-benchmarks).
Data analysis	Study data analysis code is provided in ehr_feat repository: https://bitbucket.org/hermanlab/ehr_feat. The FEAT method (v0.4.2) is in a dedicated repository: https://github.com/cavalab/feat. MIMIC-III analysis code can be found at https://github.com/cavalab/mimic3-benchmarks.

For manuscripts utilizing custom algorithms or software that are central to the research but not yet described in published literature, software must be made available to editors and reviewers. We strongly encourage code deposition in a community repository (e.g. GitHub). See the Nature Portfolio guidelines for submitting code & software for further information.

Data

Policy information about availability of data

All manuscripts must include a data availability statement. This statement should provide the following information, where applicable:

- Accession codes, unique identifiers, or web links for publicly available datasets
- A description of any restrictions on data availability
- For clinical datasets or third party data, please ensure that the statement adheres to our policy

The PMLB data is available from https://github.com/EpistasisLab/pmlb. MIMIC-III data is available from https://physionet.org/content/mimiciii/1.4/. The other EHR data in this article cannot be shared publicly to protect the privacy of the subjects. However, upon request and subject to appropriate approvals, it will be shared by the corresponding author.

Human research participants

Policy information about studies involving human research participants and Sex and Gender in Research.

Reporting on sex and gender	Subject sex, as documented in the EHR, is used in this study. Data was extracted prior to the institution of more precise clinical annotation of sex and gender. So, while the intention and label is sex, it may sometimes reflect gender. This data element was available as a binary indicator for prediction modeling.
Population characteristics	The mean age of subjects was 57.2 (SD: 18.5) years old. Subjects' sex was indicated as female for 737 (61%) and as male for 462 (39%). Race was indicated as 338 (28%) Black, 446 (37%) White, and 108 (9%) other or unknown.
Recruitment	Subjects were included for study based on evidence of longitudinal, primary care at Penn Medicine. This included 5 outpatient encounters in at least 3 distinct years between 2007 and 2017, as well as two encounter at one of 40 primary care practices. The cohort included 1000 subjects randomly selected from this cohort and 200 subjects positive for preliminary and final heuristics for treatment-resistant hypertension or hypertension with unexplained hypokalemia.
Ethics oversight	University of Pennsylvania Institutional Review Board.

Note that full information on the approval of the study protocol must also be provided in the manuscript.

Field-specific reporting

Please select the one below that is the best fit for your research. If you are not sure, read the appropriate sections before making your selection.

☒ Life sciences ☐ Behavioural & social sciences ☐ Ecological, evolutionary & environmental sciences

For a reference copy of the document with all sections, see nature.com/documents/nr-reporting-summary-flat.pdf

Life sciences study design

All studies must disclose on these points even when the disclosure is negative.

Sample size	No formal sample size calculations were performed for the primary analyses. The primary goal of this report was to assess methods rather than specific models. To help readers interpret the results, analytical results were reported with confidence intervals and p-values. The size of the cohort was decided upon empirically by observing saturation of model performance metrics with increasing sample size.
Data exclusions	Data from one subject whose clinical patient identifier changed during the course of the study was excluded.
Replication	(1) Methods were first evaluated using a set of PMLB benchmark datasets. (2) Methods were then assessed in Penn EHR data using 5-fold cross-validation within the training set. (3) Methods were applied to train models using the entire training set and evaluated on a held-out testing set. (4) Methods were applied in an internal-external design in which models were trained in patients from a specific subset of primary care practice sites and then evaluated in patients from a distinct set of practices. (5) Methods were then applied to MIMIC-III data.<br><br>There was no evidence of failure of replication.
Randomization	Samples were pre-split into training and testing datasets. These included subjects ascertained randomly and those ascertained based on heuristic-positivity.
Blinding	Investigators were not blinded to phenotypes. To account for the potential influence of outcome data on the assessment of method performance, we strictly held out separate testing data sets. The test sets were only accessed after the method refinement was complete.

Reporting for specific materials, systems and methods

We require information from authors about some types of materials, experimental systems and methods used in many studies. Here, indicate whether each material, system or method listed is relevant to your study. If you are not sure if a list item applies to your research, read the appropriate section before selecting a response.

Materials & experimental systems

n/a	Involved in the study
☒	☐ Antibodies
☒	☐ Eukaryotic cell lines
☒	☐ Palaeontology and archaeology
☒	☐ Animals and other organisms
☐	☒ Clinical data
☒	☐ Dual use research of concern

Methods

n/a	Involved in the study
☒	☐ ChIP-seq
☒	☐ Flow cytometry
☒	☐ MRI-based neuroimaging

Clinical data

Policy information about clinical studies

All manuscripts should comply with the ICMJE guidelines for publication of clinical research and a completed CONSORT checklist must be included with all submissions.

Clinical trial registration	As this was not a clinical trial, it was not registered.
Study protocol	There was no full study protocol.
Data collection	There were no patients recruited in this study. Retrospective data was collected from Penn Medicine electronic health record. Patients were ascertained based on encounter data between 2007 and 2017. Data were included from between 1997 and 2017.
Outcomes	The primary outcome for the apparent treatment-resistant hypertension prediction model was positive predictive value of >= 0.70. Secondary outcomes included sensitivity, interpretability, area under the precision-recall curve, and area under the receiver-operating curve.
